# Neuroinflammation and Cytokines in Myalgic Encephalomyelitis/Chronic Fatigue Syndrome (ME/CFS): A Critical Review of Research Methods

**DOI:** 10.3389/fneur.2018.01033

**Published:** 2019-01-10

**Authors:** Michael B. VanElzakker, Sydney A. Brumfield, Paula S. Lara Mejia

**Affiliations:** Division of Neurotherapeutics, Massachusetts General Hospital, Harvard Medical School, Boston, MA, United States

**Keywords:** myalgic encephalomyelitis, neuroimaging, glia, microglia, PBR28, cytokines, translocator protein, positron emission tomography

## Abstract

Myalgic encephalomyelitis/chronic fatigue syndrome (ME/CFS) is the label given to a syndrome that can include long-term flu-like symptoms, profound fatigue, trouble concentrating, and autonomic problems, all of which worsen after exertion. It is unclear how many individuals with this diagnosis are suffering from the same condition or have the same underlying pathophysiology, and the discovery of biomarkers would be clarifying. The name “myalgic encephalomyelitis” essentially means “muscle pain related to central nervous system inflammation” and many efforts to find diagnostic biomarkers have focused on one or more aspects of neuroinflammation, from periphery to brain. As the field uncovers the relationship between the symptoms of this condition and neuroinflammation, attention must be paid to the biological mechanisms of neuroinflammation and issues with its potential measurement. The current review focuses on three methods used to study putative neuroinflammation in ME/CFS: (1) positron emission tomography (PET) neuroimaging using translocator protein (TSPO) binding radioligand (2) magnetic resonance spectroscopy (MRS) neuroimaging and (3) assays of cytokines circulating in blood and cerebrospinal fluid. PET scanning using TSPO-binding radioligand is a promising option for studies of neuroinflammation. However, methodological difficulties that exist both in this particular technique and across the ME/CFS neuroimaging literature must be addressed for any results to be interpretable. We argue that the vast majority of ME/CFS neuroimaging has failed to use optimal techniques for studying brainstem, despite its probable centrality to any neuroinflammatory causes or autonomic effects. MRS is discussed as a less informative but more widely available, less invasive, and less expensive option for imaging neuroinflammation, and existing studies using MRS neuroimaging are reviewed. Studies seeking to find a peripheral circulating cytokine “profile” for ME/CFS are reviewed, with attention paid to the biological and methodological reasons for lack of replication among these studies. We argue that both the biological mechanisms of cytokines and the innumerable sources of potential variance in their measurement make it unlikely that a consistent and replicable diagnostic cytokine profile will ever be discovered.

## Introduction

Chronic fatigue syndrome (CFS) is an often-debilitating illness that can feel like an ongoing flu that lasts for years. Symptoms include reduced energy production, body aches, non-refreshing sleep, and difficulty recovering from both physical and mental exertion. Among many patients and some scientists, the preferred name for chronic fatigue syndrome is *myalgic encephalomyelitis* (ME), leading this condition to frequently be referred to as ME/CFS (among some scientists, the preferred name is “systemic exercise intolerance syndrome” [SEID; (1)] but use of this term remains rare). While it is more commonly used in Europe, the term “myalgic encephalomyelitis” is almost unheard of in the United States outside of experts and advocates, and “chronic fatigue syndrome” is generally used instead. The current review is largely centered on some of the research methods necessary for justifying the term “myalgic encephalomyelitis,” which essentially means “muscle pain (*myalgia*) related to central nervous system inflammation (*encephalomyelitis*).”

For this condition to warrant the name ME, “encephalomyelitis” should be a consistent finding reported by multiple groups using multiple methods. To move past a defensive posture of “*this is a real condition with biological differences from healthy controls”* toward diagnostic biomarkers and effective treatment options, the field's neuroimmunology research must be able to answer:

- How would a measured component of neuroinflammation lead to symptoms?
- How do we accurately measure that component of neuroinflammation?
- What can and cannot be concluded from the chosen method?

In this review, we focus on three specific methods that have been used to study the neuroimmunology of ME/CFS:

- positron emission tomography (PET) using translocator protein (TSPO) binding radioligand,
- magnetic resonance spectroscopy (MRS), and
- assays measuring cytokines in blood and cerebrospinal fluid

We offer a particular focus on what can and cannot be concluded by studies using these methods.

We review the above three methods because:

1. we believe that PET scanning using TSPO-binding radioligand is the best-available and most direct option for studies of neuroinflammation but that methods must be optimized,
2. MRS is much more widely available than PET with TSPO-binding radioligand and has good potential for a less expensive and invasive option for indirectly imaging neuroinflammation, and
3. studies commonly seek to find a distinct peripheral circulating cytokine “profile” in ME/CFS, and we offer critiques of current approaches.

### “Encephalomyelitis”

There have been scores of historical outbreaks of viral-like illnesses that lead to profound and lasting fatigue, perhaps most famously in Los Angeles (1934), Iceland (1948), London (1955), and Nevada (1984) (2–5). In 1955, an Icelandic doctor suggested the name “benign myalgic encephalomyelitis” after noting some similarities in cerebrospinal fluid abnormalities between patients from the London Royal Free Hospital outbreak and other putatively similar outbreaks, including a 1948 outbreak in Akureyri, Iceland (Sigurdsson May 26, 1956, in *The Lancet*). A lack of consistent methods and cerebrospinal fluid sample sizes precluded strong conclusions about similarities, or lack thereof, across the outbreaks. Sigurdsson (2) described “symptoms and signs of damage to the brain and spinal cord, in a greater or lesser degree” and “protracted muscle pain with paresis and cramp” in explaining his choice of the term “benign myalgic encephalomyelitis.” The term “benign” was included not because the symptoms were mild, but rather for discriminant validity because this “new clinical entity” was believed by Sigurdsson to have a “relatively benign outcome” (including lack of fatalities), relative to possibly similar conditions such as poliomyelitis. Another seemingly similar outbreak occurred in 1984–5 in Incline Village, Nevada. If there existed any connection to previous outbreaks that connection was not made, and a new term, “chronic fatigue syndrome,” was coined. This has contributed to confusion over whether “chronic fatigue syndrome” and “myalgic encephalomyelitis” are the same entity. The causes of and connections among outbreaks remain incompletely understood.

Despite the issues with name and diagnosis, there may be a core/root condition “ME/CFS” that involves inflammation of the central nervous system. Many studies, including those reviewed below, have reported results consistent with a neuroinflammatory process [e.g., (6–9)]. However, despite some cases of direct evidence and a fair amount of indirect evidence from case-control studies, consistent and well-replicated direct evidence for nervous system inflammation is still somewhat limited, relative to what one would expect for a condition named after a mechanistic trait.

## Inflammation Neurocircuitry

Many patients with ME/CFS report having experienced a viral or bacterial infection directly prior to the onset of their illness [e.g., (10–14)]. This has led researchers to investigate the hypothesis that resulting inflammation may be a mechanism by which this syndrome occurs [e.g., (9); (6)]. Given the putative centrality of neuroinflammation in ME/CFS, dysregulation in peripheral immune system to nervous system inflammation pathways should be a target for hypotheses and research [e.g., (15)].

When an inflammatory response occurs in the periphery, the brain is alerted to the presence of inflammation-associated molecules such as proinflammatory cytokines circulating in blood. While new potential neuroimmune pathways are still being discovered [e.g., (16)], we know of three ways in which this alert can occur. Immune proteins such as cytokines will:

1. be actively transported across the blood-brain barrier (BBB),
2. passively diffuse through the BBB via circumventricular organs if present in high enough concentrations, or
3. be detected by chemoreceptors in the afferent (sensory) vagus nerve, which synapses in the nucleus of the solitary tract (NTS) of dorsal brainstem (17–21).

The process of afferent neuroimmune signaling triggers the *sickness response* (sometimes called sickness behaviors), a general innate immune system reaction [e.g., (22)] that includes many symptoms that overlap with ME/CFS symptoms [e.g., (15)].

Cytokine signaling from the peripheral side of the BBB triggers a “*mirror response”* of glial activation and cytokine release on the brain side of the BBB (18). Glia are a class of cells that function at the intersection of the nervous and immune systems; the primary glia of the central nervous systems are *microglia*, tissue-resident macrophages that are capable of detecting danger-associated molecules such as alarmins and mitochondrial DNA, or immune signaling molecules such as chemokines and proinflammatory cytokines (23). When this detection occurs, microglia and other glial cell types enter a functional and morphological state of *activation*, and in turn produce their own chemokines and proinflammatory cytokines that can cause the activation and proliferation of nearby glia. Importantly, a relatively large brain-side “mirror response” of glial activation and cytokine release can be triggered by a small quantity of proinflammatory cytokine, if that small quantity of cytokine has been detected by the chemoreceptors of the afferent vagus nerve. Mirror responses may follow specific neural circuits (discussed below), as glia are most dense along white matter tracts (24, 25). This explains why, from the above-described three mechanisms of cytokine-to-brain communication, neuroimmune signaling continues along specific brain pathways. *Basic neuroimmunology research has begun to elucidate these pathways, which should be the focus of ME/CFS neuroimaging studies*. Kraynak et al. (19) conducted a useful meta-analysis of this basic neuroimmunology research. They synthesized results from studies that performed neuroimaging during peripheral immune activation by either an immune stimulating antigen (e.g., lipopolysaccharide [LPS]) or proinflammatory cytokines (e.g., interferon alpha [IFN-α]). Such challenges consistently activated known intrinsic brain networks and specific structures. Consistent activation occurred in basal ganglia (bilateral striatum), limbic structures (right amygdala, bilateral hippocampus, and hypothalamus), brainstem/pons, and neocortex (right anterior insular cortex, right temporal and left parahippocampal gyri, subgenual and dorsal anterior cingulate cortex [sgACC and dACC], and dorsomedial and ventromedial prefrontal cortex [dmPFC and vmPFC]). The meta-analysis also investigated functional connectivity patterns among the above structures, finding especially strong connectivity between brainstem and right anterior insula, anterior insula, and amygdala/parahippocampal gyrus, and between brainstem and sgACC/vmPFC.

Though not as robust, right temporal and left parahippocampal gyri also showed significant functional connectivity with the above structures. Therefore, these could be considered a priori functional circuits of interest in studies of putative neuroinflammatory conditions such as ME/CFS. The dACC (which would be considered anterior midcingulate cortex [aMCC] by some anatomists) did not show functional connectivity with the above circuits but was consistently activated and therefore could also be considered an a priori region of interest in neuroinflammation studies. Given the role of dACC in attention and cognitive control, we suggest that its function in ME/CFS could be considered particularly important for “brain fog” symptoms. Furthermore, Kraynak et al. (19) reported that the thalamus was also consistently detected across multiple study designs, but not in a way that demonstrated functional connectivity. However, we consider thalamus an important region of interest in ME/CFS given its detection by Nakatomi et al. (8) and given the role of thalamus in sensory filtering, a likely mechanism for the common symptom of sensory sensitivity (discussed further in section **MRS studies in ME/CFS**).

**Figure 1:**
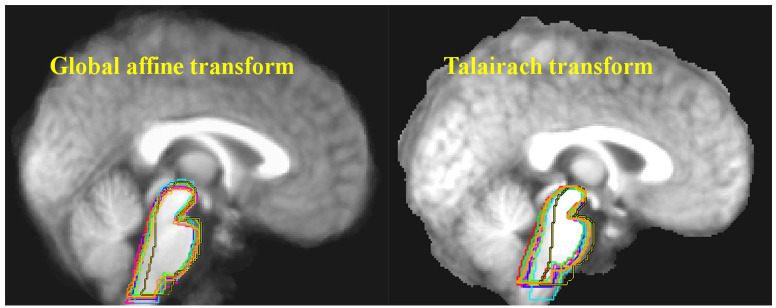
Ten structural MRI scans were aligned using two different standard neocortex-based spatial registration techniques. The brainstem of each individual brain was then traced to demonstrate how poorly they are aligned by these methods. In functional neuroimaging, detection of activation in a given brain structure is completely dependent upon the alignment of that structure across all subjects. No signal will be detected if the region of interest is not aligned. Reprinted from Napadow et al. (29) with permission from Elsevier.

In brainstem/pons, the meta-analysis did find functional connectivity but failed to find consistent activation across studies in the area of nucleus of the solitary tract (NTS) and area postrema. This might be considered unexpected because these neighboring structures are central to two of the three cytokine-to-brain pathways described in section **Inflammation neurocircuitry**: the NTS is where vagus nerve enters the brainstem, and area postrema is a key circumventricular organ. We suspect that the area of NTS and area postrema was not consistently activated in all studies of this meta-analysis because *most neuroimaging studies do not use brainstem-specific spatial registration techniques* (discussed in more detail below in section **Brainstem-specific analyses and techniques**). We therefore strongly recommend that neuroimaging studies of ME/CFS consider this area (at the dorsal surface of brainstem just inferior to pons) as an a priori region of interest. In addition to its role in afferent cytokine-to-brain signaling, this area of brainstem may hold particular importance for ME/CFS symptoms. In the afferent direction, area postrema is dense with mast cells (26), which is perhaps important for some ME/CFS patients, given comorbidity between ME/CFS and mast cell activation disorder. In the efferent direction, this area includes the dorsal motor nucleus of the vagus nerve (DMV), which is potentially important given its role in autonomic functions [e.g., (27)] that are dysfunctional in ME/CFS, such as appropriate heart rate adjustments to postural changes and exertion. Furthermore, an efferent signal from DMV should trigger an anti-inflammatory reflex, which serves to limit the inflammatory response (28). Functional analysis of this area critically relies upon brainstem-specific techniques (see Figure 1) in order for signal to be detected (27, 29).

Because neuroinflammation can affect normal function and structure, even methods that do not directly measure neuroinflammation (e.g., fMRI and structural MR) can be clarifying if their focus is on neuroinflammation-relevant brain circuits and structures. However, there are neuroimaging techniques that can more directly measure neuroinflammation, such as PET and MRS. The current gold standard for *in vivo* imaging of neuroinflammation is PET scanning using a translocator protein-binding radioligand.

## Measuring Microglial Activation: PET and The Translocator Protein

Positron emission tomography (PET) is a neuroimaging method that involves the injection of a radioactive tracer (radiotracer). The radiotracer is biologically relevant in some manner; for example it may mimic endogenous glucose or an endogenous neurotransmitter, or it may bind to a receptor or other molecule of interest. Radiotracers typically use a small amount of rapidly-decaying radiation, and as its radiation decays its location within the body or brain is calculated by the PET scanner. This allows neuroscientists to determine where the biological process of interest is occurring. Several radiotracers have been developed to detect and localize microglial activation by binding to the translocator protein [usually referred to as TSPO but also sometimes referred to as TP-18; reviewed in (30–32)].

First known as the peripheral benzodiazepine receptor (PBR), what is now called the 18kD **translocator protein (TSPO)**, is part of a larger protein complex known as mitochondrial permeability transition pore (MPTP). TSPO is expressed by non-neuronal cells of the central nervous system, and is mostly localized to the outer mitochondrial membrane. TSPO is of interest in the functional imaging of neuroinflammation because it is produced when microglia become activated, and microglial activation is a key component of classically-defined neuroinflammation. Importantly for its use as a proxy for neuroimmune functional state, TSPO is not highly expressed by microglia at a constitutive level but is upregulated upon microglial activation.

Some researchers argue that *microglial activation* is not a perfect synonym for *neuroinflammation* and that classically-defined inflammation is when circulating immune cells penetrate into tissue [e.g., (21)]. However, microglial activation would be a predictable correlate to *classically-defined neuroinflammation*, which would be defined as the infiltration into brain parenchyma of peripheral immune cells such as T cells, dendritic cells, and peripheral mast cells (18). Microglial activation is central to the increased permeability of the BBB that is necessary for this process, and therefore the binding of radiotracer to TSPO is an expected state during classically-defined neuroinflammation, and an absence of such binding would be fairly good evidence for a lack of classically-defined neuroinflammation. Other expected changes during classically-defined neuroinflammation would include activation of other resident immunocompetent cells in addition to microglia (such as astrocytes), disruption of BBB, penetration of peripheral immune cells to the brain side of the BBB, and additional possible pathological consequences such as cell loss, iron accumulation, and edema. Each of these expected changes can be measured with neuroimaging [for review of methods see (33)] and such studies would provide concurrent validity for TSPO-binding radioligand studies. Here we describe one PET TSPO study of healthy individuals, and one of individuals with ME/CFS.

### PET Scanning Using TSPO-Binding Radioligand in Healthy Humans

Sandiego et al. (34) used PET scanning with TSPO-binding radioligand to understand the effects of a peripheral immune challenge on the brains of healthy humans. They used lipopolysaccharide (LPS, sometimes called endotoxin), a molecule found in the outer membrane of gram-negative bacteria, which triggers an immune response via TLR4 signaling. LPS is a commonly-used experimental immune challenge, but the effect of peripheral LPS injection on immune response in the brain had previously only been studied in animal models.

Using a within-subjects design, Sandiego et al. (34) reported significantly increased PBR28 signal in many brain structures following LPS-injection, including bilateral caudate nucleus and putamen of the basal ganglia, large areas of the neocortex, amygdala, hippocampus, and thalamus. They also found a significant increase in several peripherally-circulating cytokines; however, these circulating cytokine levels correlated with neither PBR28 signal nor subjective sickness symptoms such as fatigue. This is an important concept found repeatedly in the neuroinflammation literature and discussed further below: *circulating cytokine levels are often a poor measure for subjective symptoms and often do not reflect what is happening on the brain side of the BBB*. For example, in a study of ME/CFS, Nakatomi et al. (8) reported a lack of correlation between circulating cytokine levels and TSPO-binding radioligand signal in ME/CFS patients' brains, along with a lack of correlation between circulating cytokine levels and their subjective symptoms.

### PET Scanning Using TSPO-Binding Radioligand in ME/CFS

Nakatomi et al. (8) conducted the first case-control study using PET to measure TSPO expression in the central nervous system of ME/CFS patients vs. healthy controls. They found significantly increased PET signal, especially in a region between mid-pons and thalamus, in patients vs. controls. Based on how the “mirror response” of peripheral-to-central nervous system immune signaling works, this is the general pattern one would expect based on a paper from our group, which hypothesizes that some cases of ME/CFS could be explained by exaggerated afferent neuroimmune signaling entering the central nervous system at the nucleus of the solitary tract (NTS) in dorsal brainstem (15). Nakatomi et al. (8) remains an important, groundbreaking study that should be replicated with complementary methods. Here, we describe several specific ways to complement and improve upon future studies using the same general method of PET scanning using TSPO-binding radioligand.

### Methods to Address Potential Confounds in PET Studies Using TSPO-Binding Radioligand

There are several potential ways to interpret differences in TSPO-binding radioligand signal in patients vs. controls. Isolating and addressing potential confounding variables will make interpretation easier but also adds difficulty and considerable cost to a study. Type 1 or type 2 errors in studies of TSPO-binding PET radioligand uptake in brain could potentially be explained by the following methodological confounds:

- Standard neuroimaging techniques were not designed for brainstem study
- The first-generation radioligand PK11195 has high non-specific binding and low signal-to-background ratio
- PET signal calculated with an anatomical reference brain region relies on equal radioligand uptake in that region across cases and controls
- Radioligand access to brain is modified by general metabolism, which can differ across cases and controls
- Activated peripheral immune cells bind radioligand and can differ in quantity across cases and controls
- A single nucleotide polymorphism (SNP) in the TSPO gene causes differential radioligand binding
- Use of healthy controls harms discriminant validity

Here, we will address each of these issues and describe solutions.

#### Brainstem-Specific Analyses and Techniques

##### Standard neuroimaging techniques were not designed for brainstem study

One can almost consider the structural and functional neuroimaging analysis of brainstem to be a separate technique from the analysis of neocortex because brainstem analysis has its own issues that must be resolved for the data to be interpretable [e.g., (27, 29, 35)]. The vast majority of neuroimaging studies do not use brainstem-appropriate techniques. Two prominent issues are (1) the need for independent spatial registration of brainstem, and (2) the unique susceptibility of brainstem to physiologically-based movement artifact.

Standard MRI and fMRI analysis software platforms use the neocortex for spatial registration. In neuroimaging, spatial registration is the process of lining up all participant brains so their anatomy overlaps, allowing structural differences or functional activations to be meaningfully compared. Nakatomi et al. (8) used Statistical Parametric Mapping 5 software (SPM5; Wellcome Department of Cognitive Neurology), which is a well-validated and widely-accepted technique in neuroimaging. However, like most standard techniques, the brainstem is not the focus of standard SPM5 spatial registration. Instead, the neocortex of each individual brain in a study is lined up with the neocortex of a canonical brain (see Figure 1). This is because the vast majority of functional neuroimaging studies examine the types of “higher” cognitive and emotional processes that are associated with the neocortex, as opposed to studying the types of “lower” processes that are associated with the brainstem (e.g., autonomic, arousal, pain, neuroimmune communication). Given the anatomical reality that the brainstem comprises many densely-packed but functionally-heterogeneous nuclei, any small errors in spatial registration caused by failure to use brainstem-specific registration are highly likely to lead to decreased sensitivity in signal and type 2 errors (29). It is likely a testament to the strength of the PET signal in Nakatomi et al. (8) that their results remained statistically significant despite the fact that brainstem-specific analysis techniques were not used, however it is also likely that the lack of dorsal signal is explained by this confound.

Furthermore, the brainstem is especially prone to physiologically-driven movement artifact given that it pulses with every heartbeat [e.g., (36, 37)]. This is especially important for fMRI studies as opposed to PET, but this artifact is rarely considered in studies using either method. This can be corrected by recording physiological measures during acquisition to use as a movement artifact regressor during functional analysis. It is important to note that failure to control for systematic differences in movement between patients and controls has caused significant confusion in some clinical neuroimaging fields [e.g., (38)].

A large majority of neuroimaging studies in ME/CFS have not used brainstem-specific spatial co-registration, normalization, or physiologically-derived movement artifact regression techniques. In a disorder defined by symptoms related to fatigue, autonomic nervous system problems [e.g., (39–46)], and putative neuroimmune signaling [e.g., (6, 9, 15)], brainstem is an obvious region of interest. Standard analysis techniques would surely fail to coregister the very small nuclei that may be related to key ME/CFS symptoms (e.g., nucleus of the solitary tract, area postrema, dorsal motor nucleus of the vagus nerve, periaqueductal gray, reticularis gigantocellularis, and others). It is therefore quite likely that functional brainstem abnormalities in this condition, if any, have been missed by those studies that reported the results of standard techniques. It is noteworthy that several studies that did deliberately focus on brainstem have found abnormalities. For example, Costa et al. (47) reported brainstem hypoperfusion in ME/CFS patients vs. depressive and healthy controls. Barnden et al. (48) reported differential regression values of seated pulse pressure (systolic–diastolic) against brainstem total gray matter volume (measured by voxel-based morphometry and centered on tegmental area) in ME/CFS patients vs. healthy controls. Similarly, Barnden et al. (39) reported an abnormal association between indicators of autonomic function volumetric measures in the area of the vasomotor center in the brainstem's medulla oblongata, which (along with glossopharangeal nerve) is innervated by the neuroimmune and autonomic parasympathetic vagus nerve. Barnden et al. (49) reported abnormal T1-weighted spin echo MRI signal in brainstem of Fukuda criteria ME/CFS patients.

By using brainstem-specific spatial registration in addition to standard neocortex spatial registration, neuroimaging studies of ME/CFS are much more likely to detect any functional and structural abnormalities that may be driving autonomic and neuroimmune-related symptoms. We believe it likely that failure to use brainstem-specific techniques has resulted in type 2 errors in the ME/CFS neuroimaging field, in PET studies as well as other modalities like MRI and fMRI.

#### PBR28 or Other Second-Generation Radioligands Instead of PK11195

##### The first-generation radioligand PK11195 has high non-specific binding and low signal-to-background ratio

Nakatomi et al. (8) used the first-generation TSPO-binding radioligand, [11C]-(R)-PK11195 (referred to hereafter as PK11195). The development of PK11195 in the 1980s led to advances in the understanding of brain diseases with an inflammatory component such as multiple sclerosis, Rasmussen's encephalitis, Huntington and Alzheimer's diseases, and others (31). However, PK11195 has fairly low brain penetrance and also high non-specific binding in that it binds to other types of immune cells and proteins, including those in the general blood circulation [e.g., (50)]. If there are systematic differences in BBB permeability or in the quality and quantity of PK11195-binding antigens between cases and controls, this can lead to type 1 or type 2 error. Since PK11195's development, a newer, second-generation family of TSPO-binding radioligands has been created (33). Second-generation TSPO-binding radioligands, including PBR28, FEPPA, and DPA-714 [reviewed in (51)], feature a much higher signal-to-background ratio than PK11195.

#### Arterial Line (A-Line) Sampling During PET Neuroimaging Allows Data Interpretation

##### PET signal calculated with an anatomical reference region relies on equal radioligand uptake in that region across cases and controls

Nakatomi et al. (8) used a cerebellar reference region to calculate non-displaceable binding potential: in order to compare patients to controls, each individual study participant had the amount of PET signal in brain regions of interest compared to the amount in the cerebellum. In other words, each person's cerebellum was used as their own “baseline” comparator to decide if other regions were showing evidence of radioligand uptake and therefore microglial activation. This is a standard and widely-accepted technique for PET study analyses, however it is not a quantitative analysis technique: the “signal” reported in such studies is a relative signal and not a quantitative one. This may be particularly important for studies of a poorly-understood condition like ME/CFS because we cannot be certain that the cerebella of patients are not affected by their condition. For example, cerebellar folia (gyri) contain several large blood vessels which could contain different amounts of TSPO-expressing circulating immune cells in patients vs. controls. Furthermore, a recent report found increased HHV-6 infection of cerebellum Purkinje cells in mood disorders vs. controls (52); such an infection would be likely to increase TSPO expression and render invalid the cerebellum as a “baseline” reference region. The gold standard for quantitative data would be arterial line (A-line) sampling for kinetic modeling of TSPO, which counters other potential confounds as well. Throughout the scan, blood samples are extracted from the radial artery at regular timepoints. Sample analysis allows determination of the exact quantity of free radioligand available to enter the brain, which is used to interpret brain signal.

##### Radioligand access to brain is modified by general metabolism, which can differ across cases and controls

One common theory of ME/CFS is that it is, at root, a disorder of mitochondrial dysfunction and reduced metabolism [e.g., (53, 54)]. This creates a possible alternative explanation for the increased PK11195 uptake demonstrated in Nakatomi et al. (8). If metabolism is reduced in ME/CFS patients relative to healthy controls, the radioligand would be metabolized more slowly in patients. This means that more radioligand would reach the brain for the simple reason that more remains circulating from the original injection. This problem is made worse by low-brain-penetrance radioligand such as PK11195 as opposed to second-generation radioligands such as PBR28. The use of A-line sampling during scanning can provide an ongoing measure of arterial radioligand availability, allowing any individual differences in radiotracer metabolism to be taken into account.

##### Activated peripheral immune cells bind radioligand and can differ in quantity across cases and controls

While PBR28 has improved non-specific binding, the antigen that it binds to can occur in non-target tissues and in blood. Neurologists, neuroimmunologists, and neuroscientists use PET radioligands that bind to TSPO because TSPO is produced by activated microglia, the resident tissue macrophages of the central nervous system. However, there are many different kinds of tissue macrophages as well as macrophages in general circulation, and these cells also produce TSPO. Many medical conditions are associated with changes in TSPO expression within different peripheral organs [e.g., (55–58)]. Use of an A-line protects against the possibility that group differences in circulating cells, molecules, and tissue macrophages (possibly due to comorbid conditions) cause differences in peripheral TSPO binding, thereby leaving less TSPO-binding radioligand capable of reaching the brain.

#### Genetic Analysis of the TSPO Gene

##### A single nucleotide polymorphism (SNP) in the TSPO gene causes differential radioligand binding

*In vitro* studies demonstrate that PK11195 and second-generation TSPO-binding radioligands have different binding sites on the TSPO protein (59). The gene for TSPO (*Ala147Thr*) can have different polymorphisms, including the rs6971 SNP which significantly explains the binding affinity of second-generation TSPO PET radioligands (60, 61). The literature has therefore described a trimodal binding affinity distribution in terms of high-affinity binder (HAB), low-affinity binder (LAB), and mixed affinity binder (MAB) subjects. Because they used PK11195, which binds to a different site on TSPO, Nakatomi et al. (8) did not need to report genetic analysis of the rs6971 SNP. Practically speaking, it is unlikely that replication efforts would be confounded due to the accidental recruitment of all HAB patients and all LAB controls but this is a potential confound that must be ruled out. Therefore, all efforts to replicate and expand upon the pioneering work of Nakatomi et al. (8) should report genetic analyses.

#### Control Group Selection and Discriminant Validity

An important goal for the ME/CFS field is to find objective biomarkers for both symptom severity and diagnosis; TSPO as measured by PET radioligand binding is one such potential biomarker. Diagnostic biomarkers must show discriminant validity, that is (assuming for a moment that ME/CFS is one entity), they must be able to differentiate ME/CFS from other medical conditions. Nakatomi et al. (8) reported increased PK11195 signal in ME/CFS patients relative to healthy controls, as opposed to mechanistically relevant disease conditions or sedentary controls. An important consideration is that PET studies have shown increased TSPO radioligand uptake in many different neurological and psychiatric conditions, such as autism, traumatic brain injury, major depression, bipolar disorder, Parkinson's disease, chronic pain, multiple sclerosis, and schizophrenia [e.g., (62–66)]. This represents another reason to include non-healthy control groups in studies of putative ME/CFS biomarkers. Furthermore, there is some evidence from a rodent model that translocator protein radioligand uptake may be influenced by exercise (67), which is another argument for the importance of including sedentary controls in studies of ME/CFS. The use of sedentary-matched controls is an important consideration in all studies for which it is possible, not just PET scan studies.

## Magnetic resonance spectroscopy (MRS) in neuroinflammation

### MRS Can Complement PET for Studying Neuroinflammation

PET is a highly sensitive neuroimaging method, capable of detecting very subtle biological changes that would be missed by other imaging modalities. PET is also capable of quantifying specific neuroinflammation-relevant biological targets such as TSPO. However, there are also multiple downsides to this method, some of which are not present with magnetic resonance spectroscopy (MRS), a neuroimaging technique that uses the MRI modality. Like PET, MRS is capable of measuring the concentration of specific biochemicals. We discuss the mechanisms of MRS here, followed by some of the relative advantages and disadvantages of MRS vs. PET in the study of neuroinflammation.

MRS can measure the relative concentrations of a variety of biochemicals, often referred to in the MRS literature as “metabolites.” This can be accomplished with a powerful magnet because chemicals vary in the density of electrons surrounding their nuclei. Therefore, a strong magnetic field “bounces” back from each metabolite in a signature way, and this can be measured by the MR computer: differences in the reflected magnetic fields can be converted into a readable output spectrum. MRS methods are currently capable of detecting a few dozen metabolites with known spectral properties, and MRS researchers choose from this list of metabolites when designing their analyses. After an a priori decision to focus on a particular part of the spectrum, metabolites are generally reported as a ratio (one metabolite vs. another reference metabolite) as opposed to an absolute concentration. These are among the reasons that MRS is not nearly as sensitive or specific as quantitative measurement of PET radioligand uptake, but there are also some ways in which MRS has advantages over or can complement PET when they are acquired together.

PET is somewhat invasive because PET radioligands must be injected; patient discomfort can increase if an arterial line is used for quantitative measurement. MRS, on the other hand, requires neither an injection nor radiation. Largely because PET radioligands have a short radioactivity half-life, they must be made on-site or near imaging facilities. This is a limiting factor especially for radioligands that are not yet approved for clinical use, because most hospitals with PET scanners would not have access to experimental radioligands. These are among the reasons PET studies are generally more than twice as expensive as studies using MRI-based methods such as MRS. Furthermore, due to the radiation involved in PET procedures, only a limited number of research scans per year are allowed for each participant, whereas there is no such limitation for MRI or MRS scanning. Relatedly, study recruitment can be more difficult when a protocol calls for an injection of radioligand or an A-line. A small number of facilities have access to dual MR-PET scanners, which can combine modalities in a single scanning session (68, 69). This can allow the discovery of MRS correlates to sensitive PET signal. As an example relevant to ME/CFS, in a neuroinflammatory process, one would expect both microglia and astrocytes to become activated. TSPO is produced by activated microglia but most evidence shows that it is not as strongly produced by astrocytes. MRS is capable of measuring inflammation-associated chemical changes beyond only microglial activation, including in astrocytes. With a dual MR-PET scanner, signal from MRS and TSPO-binding radioligand can be measured in the same patient at the same time, helping to better clarify the relationship between their respective neuroimaging signals.

#### Importance of a Priori Decisionmaking in MRS Studies

Similarly to how different colors occupy a different place along the visible light spectrum, MRS-detectable metabolites each occupy a different place along the magnetic resonance spectrum. However, unlike the human eye's ability to detect the entire visible light spectrum at once, MRS must be somewhat targeted to a limited window within the whole spectrum. If study participants were capable of spending unlimited time in a scanner, all metabolites could theoretically be measured in the entire brain but in reality, researchers must make thoughtful hypothesis-driven decisions about what spectra to measure and in which specific brain regions. If researchers are interested in testing the hypothesis of neuroinflammation in ME/CFS, these decisions should be based in the human neuroinflammation literature.

In some cases two metabolites almost overlap on the spectrum, while in other cases a given metabolite is quite distant from the others. Each of these scenarios presents a unique problem that must be considered before data acquisition begins. Two relatively “distant” metabolites like lactate and NAA cannot be captured with good resolution in the same scan sequence. On the other hand, glutamine, glutamate, and gamma-aminobutyric acid (GABA) are so close together that they can appear as a single peak in the MRS output unless that region of the spectrum is deliberately targeted. If a researcher is interested in understanding the relative contributions of glutamine, glutamate, and GABA, she must make that decision before the experiment begins and focus acquisition directly on the area of the spectrum where these metabolites exist. Furthermore, a priori decisions about which brain structure to measure are also important.

MRS spectra can be recorded from a “slice” of brain or from a single voxel (the 3-dimensional MRI analog to a “pixel”), each of which takes about 15–25 min to acquire. Slices cover more anatomy but have the disadvantage of including several different types of tissue within the same slice (i.e., white matter, gray matter, blood vessels, and ventricles/cerebrospinal fluid). This is a problem because the spectral signal represents an average over the measured area, and different types of tissue have different metabolite concentrations. Therefore, if multiple tissue types are in the same region, interpretation becomes difficult. With thoughtful placement, single voxel MRS has the ability to include only one tissue type, but only from a very tiny section of anatomy (e.g., 1mm^3^). The spectra recorded from slice or single voxel MRS is usually reported as a ratio of one metabolite relative to another, which can then be compared across different brain regions or in patients vs. controls.

### MRS Studies in ME/CFS

Several MRS-detectable metabolites are fairly well validated proxies for inflammation, metabolism, and brain health, and are therefore of particular potential interest for studying neuroinflammation in ME/CFS. A few studies have used MRS imaging in ME/CFS (see Table 1). These studies have looked in a wide variety of brain regions, measuring a wide variety of metabolites (70, 72–79, 81). Brief descriptions of measured metabolites are listed here.

**Table 1:** Brief review of brain magnetic resonance spectroscopy (MRS) studies in ME/CFS.

	**Study procedures**			**Metabolites (reference)**			**Specific notes**
**Study**	**Criteria used**	**Method**	**Brain region**	**Increased**	**Decreased**	**No change**	**Notes**
Natelson et al. 2017 (70)	Fukuda et al. 1994 (71)	3T Slice (280 ms)	Ventricles	Lactate			Significant between ME/CFS and control groups (not among FM only, ME/CFS only, or FM/ME/CFS groups)
Van der Schaaf et al. 2017 (72)	Fukuda et al. 1994 (71)	3T Single voxel (3.03 ms)	Dorsolateral prefrontal cortex, primary visual cortex (V1)			NAA (creatine ref)	
Shungu et al. 2012 (73)	Fukuda et al. 1994 (71)	3T Single voxel (280 ms)	Occipital cortex, ventricles	Lactate	Glutathione		Significant between ME/CFS and control groups (not versus MDD)
Murrough et al. 2010 (74)	Fukuda et al. 1994 (71)	3T Single voxel, Slice (280 ms)	Anterior cingulate cortex, occipital cortex (single voxel); ventricles (slice)	Lactate		GABA, glutamate/glutamine	Significant between ME/CFS and control groups (not versus MDD)
Puri et al. 2009 (75)	Fukuda et al. 1994 (71)	3T Single voxel (144 ms)	Cerebral cortex			Glutathione	Criteria listed as “Revised CDC Criteria”
Mathew et al. 2008 (76)	Fukuda et al. 1994 (71)	3T Slice (280 ms)	Corpus callosum, ventricles	Lactate		Choline, creatine, NAA	
Chaudhuri et al. 2003 (77)	Fukuda et al. 1994 (71)	1.5T Single voxel (1500/135 ms)	Left basal ganglia	Choline (creatine ref)	NAA (total choline ref)		
Puri et al. 2002 (78)	Fukuda et al. 1994 (71)	1.5T Single voxel (135 ms)	Left motor cortex, occipital cortex	Choline (creatine ref)		Creatine, NAA	Choline only significant in occipital cortex
Brooks et al. 2000 (79)	Holmes et al. 1988 (80)	1.5T Slice (30, 72, 144 ms)	Right hippocampus		Choline, creatine, NAA (total creatine ref)		Myo-Inositol results not reported; Choline and creatine trend-level decrease only
Tomoda et al. 2000 (81)	Holmes et al. 1988 (80)	1.5T Single voxel (500/11ms, 4000/100ms)	Frontal white matter	Choline (total creatine ref)		NAA (total creatine and total choline refs)	

*Summary of methods and results in the ME/CFS MRS literature. Method column lists MRI scanner magnet strength and MRS acquisition details (single voxel or slice; echo time). Brain region column indicates regions of interest; with the exception of lactate which is only measured in ventricles, all metabolite changes occurred in all listed regions (unless otherwise noted). Metabolites columns list changes relative to controls, with reference metabolite(s) noted in parentheses. FM, fibromyalgia; MDD, major depressive disorder; NAA, N-acetylacetate*.

**Choline** is important in the maintenance of membrane health, and therefore is a potential marker of BBB status (82). It is considered a marker for neuroinflammation because of its relationship to glial activation and BBB permeability (33).

**Creatine** is a critical regulator of energy homeostasis in the brain [e.g., (83)]. It is believed to have static levels throughout the brain of healthy individuals and is therefore often used as the standard to which other metabolites are normalized (33). Creatine and phosphocreatine are close enough on the spectrum that they are usually pooled.

**Gamma-amino butyric acid** (GABA) is an inhibitory neurotransmitter and has been linked to reduced cognitive ability (84).

**Glutamate** is the primary excitatory amino acid in the nervous system, and is produced by activated glial cells. Glutamate levels vary with a number of neurological disorders (85).

**Glutathione** is involved in the oxidative and nitrosative stress pathways as an antioxidant (86). Oxidative damage and inflammation are generally associated with low glutathione.

**Lactate** is an end-product of oxidative metabolism and is therefore a potentially interesting biomarker for a metabolism-associated illness such as ME/CFS. Lactate levels in healthy brain tissue are so low as to be almost undetectable by conventional MRS at 1.5T or 3T magnet strength, but when measured in ventricular cerebrospinal fluid, elevated lactate is associated with neuroinflammation (33, 87–92).

**Myo-inositol** is a carbocyclic sugar residing largely in astrocytes, and is upregulated during astrocyte activation (33). This makes myo-inositol a potentially interesting complement to PET scan studies that use TSPO-binding radioligand to measure microglial activation. Myo-inositol also upregulates during myelin decay (93).

**N-acetyl acetate** (NAA) production occurs in the mitochondria. Because this metabolite is found in the cytoplasm of neurons, it is considered a marker of neuronal density and therefore often used as a rough marker of brain health (82). However, NAA's normal metabolic and neurochemical functions remain incompletely understood and therefore its relationship to different disease states is controversial and complicated (94).

As evident in Table 1, there is not a clear and consistent characterization of metabolite alterations in ME/CFS. This is not because of failed replication attempts, but rather due to wide a variety of experimental designs, diagnostic criteria selection, subject populations (e.g., juvenile vs. adult), comparison control groups (e.g., healthy, fibromyalgia, or anxiety disorder), brain regions examined, and metabolites targeted.

Regarding MRS metabolite targeting: change in a given metabolite is usually reported as a ratio, relative to a chosen reference baseline. For meaningful interpretation, this requires the reference metabolite (i.e., the ratio denominator) to be stable. Due to its stability in healthy individuals, creatine is the most commonly used ratio reference metabolite, and it is the most commonly used ratio reference in studies of ME/CFS (see Table 1). However, creatine may not be ideal to use in an undercharacterized condition such as ME/CFS. Use of creatine as a ratio reference in case control studies is based on the assumption that its levels will not differ between cases and controls (i.e., interpretation of numerator changes relies upon confidence that the denominator is constant). However, creatine alterations have been reported in autism (95), a condition that may have some mechanistic (and therefore metabolite) similarities with ME/CFS. Autism, like ME/CFS, is a neuroinflammation-associated condition with large sex differences in prevalence, and sensory overload symptoms. Interestingly, an MRS study found sensory sensitivity symptoms in autism to correlate with phosphocreatine abnormalities in thalamus, a brain structure central to sensory filtering and processing (96).

Thalamus is one example of a neuroinflammation-associated a priori region of interest (19) that remains relatively understudied in ME/CFS. The choices of brain regions listed in Table 1 generally do not appear to be based in neuroinflammation-specific hypotheses. Given the fact that early MRS studies of ME/CFS have largely been exploratory, this is somewhat understandable. However, given the putative importance of neuroinflammation in this condition, we believe that the ME/CFS neuroimaging field could benefit from basing a priori targeting of brain regions of interest in the newly emerging human neuroinflammation literature recently meta-analyzed by Kraynak et al. (19), and described above. One MRS study from the human neuroinflammation literature could be a particularly important guide for a priori decisions regarding target brain regions, given the field's focus on the possible importance of peripheral proinflammatory cytokine signaling.

#### Lessons for MRS Studies of ME/CFS FROM a Study of Inflammatory Challenge in Healthy Humans

One study reviewed by Kraynak et al. (19) was Haroon et al. (97), which investigated the brain response, as measured by MRS, to injection of peripheral proinflammatory cytokine. This type of translational research seems particularly relevant to the ME/CFS field, which has long pursued evidence of neuroinflammation driven by circulating proinflammatory cytokines.

Validating a large animal literature [e.g., (98)], newer human studies have demonstrated that exogenous proinflammatory cytokines (e.g., injected IFN-α) or other immune challenges (e.g., injected typhoid vaccine) can influence behavior and fMRI brain activity in otherwise healthy humans [e.g., (99–102)]. These papers each reported increased BOLD (blood oxygen level-dependent) response in basal ganglia and dACC after challenge. However, the specific biological basis of these BOLD response alterations was not known. A clarifying question would be if brain metabolites, as measured by MRS, were also altered by exogenous proinflammatory cytokine injection or peripheral immune challenge.

IFN-α is frequently used as a treatment for hepatitis-C, and has a fairly common side effect of inducing depressive episodes or a possibly ME/CFS-relevant neurovegetative syndrome including profound fatigue (103). In order to better understand the mechanisms behind this cytokine-induced side effect, Haroon et al. (97) used MRS to investigate the effect of IFN-α injection on basal ganglia and dACC. They recruited 31 hepatitis-C virus positive individuals, who were separated into two groups: IFN-α injection vs. no injection. Both groups were assessed at baseline and again after a month. Relative to the control group, the injection group experienced increases in subjective depression and fatigue, peripheral blood inflammatory cytokines TNF and sTNFR2, and increased MRS signal for glutamate in the dACC and the left basal ganglia. *No statistically significant correlations were found between brain MRS signal and inflammatory cytokines circulating in blood*. Unfortunately, the authors did not report brainstem results and did not conduct brainstem-specific analysis. Based on this study and the Kraynak et al. (19) meta-analysis, basal ganglia and dACC are attractive a priori regions of interest in brain scan studies interested in using MRS scans to examine neuroinflammation-related changes in ME/CFS patients vs. matched controls.

## Peripheral cytokines in ME/CFS

Brain scans are expensive and require many hours of analysis before they are interpretable. Therefore, the discovery of a cheap, easy-to-obtain biomarker from peripheral blood would be an attractive alternative. One common blood measure in ME/CFS studies are *cytokines*, a broad class of inflammation-related signaling molecules comprising interferon (IFN), tumor necrosis factors (TNF), chemokines, lymphokines, and, most commonly, interleukins (IL). The ME/CFS field has pursued cytokine research in the hopes of finding a blood test that is capable of diagnosing or measuring symptom severity. Blundell et al. (104) recently reviewed this cytokine literature and explained their motivation: “Here we focus on circulating cytokines and we seek to determine whether a pro-inflammatory circulating cytokine profile exists in patients with CFS in comparison to controls and how this cytokine profile differs from controls following stimulation such as exercise.” Thus, a consistent and replicable “cytokine profile” would be a diagnostic biomarker, and further, would be evidence for an inflammatory process at the root of ME/CFS pathophysiology. However, at the conclusion of their literature review, the authors reported that they did not find a consistent “cytokine profile” in ME/CFS. In this section, we will make the argument that a lack of consistent “cytokine profile” is an inevitable result of 1) the way that cytokines actually function biologically and 2) the methods used to measure cytokines. We end with recommendations that will hopefully allow more meaningful comparisons in the future.

### Biological Mechanisms Limit the Value of Peripheral Blood Cytokines as a Stable Biomarker

Cytokines are a communication factor released by activated innate immune cells such as macrophages and mast cells in the periphery, as well as glia and endothelial cells on the brain side of the BBB. This cytokine signaling is a key component of the sickness response (22, 98), which has symptoms that overlap with key ME/CFS symptoms (15). Relatedly, cytokines are a key component of neuroinflammation; one of the key ways peripheral inflammation triggers neuroinflammation is when the vagus nerve detects peripheral cytokines [e.g., (105, 106)]. Thus, cytokines are a class of molecule that, at first blush, seem to hold promise as a potential peripheral biomarker for neuroinflammation in ME/CFS. However, the way that cytokines actually function mechanistically tarnishes some of this promise.

The core problem with looking for cytokines in peripheral blood is that cytokines generally do not function as endocrine signalers, but are rather normally autocrine and paracrine signalers (see Box 1). In other words, cytokines do not function by flowing through blood (where many studies hope to measure them due to easy access) but rather by acting locally, directly in the vicinity of infection or injury. Cytokines do not need to function as circulating endocrine molecules to drive subjective sickness symptoms because they can be detected by the sensitive and highly branched afferent vagus nerve, which communicates their presence to the brain via brainstem and triggers neuroinflammation and sickness responses (15, 105, 106). A large neuroimmunology literature consistently concludes that cytokines *do not have to be detectable in the periphery in order to have an effect on sickness-related symptoms*. For example, Campisi et al. (107) stated, “Elevated levels of circulating cytokines and endotoxin are not necessary for the activation of the sickness or corticosterone response.” Another fact of cytokine biology that makes a stable, predictable blood profile difficult is that cytokine-cytokine interactions are in constant dynamic flux and are exquisitely complicated (108), and their levels can be affected by a huge number of variables (reviewed below). Furthermore, as relatively large, lipophobic, polypeptide protein molecules, cytokines generally do not easily diffuse across an intact BBB and thus, circulating levels do not accurately reflect brain cytokine levels. Therefore, a *peripheral cytokine profile may not be meaningful in informing any existing central nervous system cytokine profile*. This general point is made by many papers in the cytokine methods literature: there is limited ability for a putative “cytokine profile” to inform underlying disease processes.

Box 1

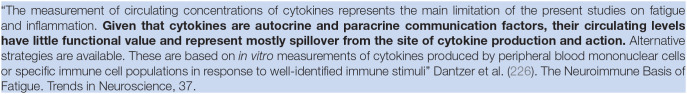

Despite the limited value of measuring blood cytokine levels in understanding pathophysiology and neuroinflammation, blood cytokine levels are used as a dependent variable in many ME/CFS studies, probably due to ease of collection. The cytokine methods literature emphasizes the need for optimization and standardization of collection, storage, and assay methods, but these factors have varied widely in ME/CFS cytokine studies. For this reason, previous studies of peripheral cytokines in ME/CFS cannot be meaningfully compared as Blundell et al. (104) set out to do.

### Cytokine Studies in ME/CFS as Reviewed by Blundell et al. 2015 (104)

The limitations of ME/CFS cytokine studies can be seen in the recent literature review by Blundell et al. (104), which aimed to “determine if a pro-inflammatory circulating cytokine profile exists in ME/CFS patients relative to controls.” Here we give a brief overview of the Blundell et al. review, and then we detail the assay methodology used in the ME/CFS cytokine literature, using the studies from the Blundell et al. review and studies published since then (see Table A1).

Blundell et al. (104) published a systematic review but were not able to conduct a conventional meta-analysis due to dissimilarities among reviewed studies. The authors began with a quality assessment, finding that 14 out of 38 reviewed studies were of poor quality due to failure to control for one or more items on a list of confounding factors that can influence cytokine levels: age, subject activity level, BMI, gender, menstrual cycle stage, comorbid diseases, antidepressant use, or diurnal variation. However, beyond those confounds, the study designs differ so much that any comparison may not be meaningful (e.g., comparing sleeping patients to exercising patients).

Despite the lack of consistent study design, the authors concluded that there is “little or no evidence to support the hypothesis that proinflammatory circulating cytokines are raised in CFS” (104). They reasoned that a failure to find consistent results across studies could be due to heterogeneity in the ME/CFS population, or due to the local rather than systemic role of cytokines in ME/CFS. While these are reasonable explanations, we would argue that the reviewed studies show such inconsistencies in cytokine measurement methods that consistent findings would be impossible even if they shared comparable research designs.

Blundell et al. (104) briefly noted the different assay types (i.e., bioassay vs. immunoassay) and sample matrices (i.e., serum vs. plasma) across studies. However, beyond these two measurement issues (and the short list of potential confounds mentioned above), a large cytokine methods literature demonstrates a staggering number of potential confounds in the measure of cytokines, with potential problems arising at every step of the way. Here, we describe the importance of additional factors in the collection, handling and processing, storage, and assaying of cytokines (detailed in Table A1).

### Methodological Confounds That Must Be Considered Before Comparing Cytokine Studies

The biological mechanisms of cytokines make a consistent and stable circulating profile unlikely, which limits the ability of peripheral cytokines to provide insight into underlying pathophysiology in ME/CFS. Therefore, a peripheral cytokine profile is unlikely to be a feasible and useful biomarker. In addition to these biological factors, there are many methodological problems.

- Even if cytokines were meaningful peripheral biomarkers: Blood cannot be compared to cerebrospinal fluid
- Even within blood sampling: Venous and arterial blood samples cannot be compared
- Even if blood sampling methods were equal: Plasma, serum, and PBMC sample matrices cannot be compared
- Even if ME/CFS researchers use a consistent sample matrix: Bioassay, ELISA, and multiplex assay results cannot be compared, even across kits of the same assay type
- Even if ME/CFS researchers standardize their methods: The same exact lab, personnel, and protocol will likely get different results from the same manufacturer's kit

The relevant details of methods used in previous studies of cytokines in ME/CFS are listed in Table A1. Importantly, this table adds to the number of factors that were listed by Blundell et al. (104) to clearly display the widespread variance of cytokine methodology in the ME/CFS literature. The intention of this section is to show that (1) currently-existing studies in the ME/CFS cytokine literature cannot be meaningfully compared due to differences in methods and (2) the ME/CFS field must consider the mechanisms of cytokines and establish some consistency in methods for any role of cytokines in ME/CFS to be elucidated.

#### Blood Cannot Be Compared to Cerebrospinal Fluid

Choosing between blood and cerebrospinal fluid is the first point of potential variability in attempts to identify a cytokine profile, as the concentrations of various cytokines are not necessarily equivalent across body fluid sample types. Most ME/CFS studies have analyzed cytokines from peripheral blood samples (see Table A1). Less frequently, others have analyzed cytokines from cerebrospinal fluid (119, 129, 177, 178). In addition to many examples of this phenomenon in the rodent literature [e.g., (179–181)], human studies have also demonstrated that cytokine concentration in cerebrospinal fluid vs. blood can differ, with some examples showing a positive correlation, some showing lack of correlation, and some showing anticorrelation [e.g., (182–186)]. Because the presence of cytokines usually reflects local rather than systemic conditions (see Box 1), measuring cytokines from the cerebrospinal fluid is a more direct representation of the central nervous system environment than from peripheral blood. Therefore, for studies interested in ME/CFS neuroinflammation, cerebrospinal fluid sampling is more likely to be useful. However, because cytokines are locally-acting paracrine and autocrine factors, one cannot assume that a sample of cerebrospinal fluid taken during a lumbar puncture spinal tap accurately reflects the entirety of the central nervous system cerebrospinal fluid. For example, Milligan et al. (179) reported IL-1 detection in cerebrospinal fluid samples taken from the lumbosacral region but not from the cervical region.

#### Venous and Arterial Blood Samples Cannot Be Compared

Because they are produced and removed in local tissues, cytokines differ in concentration between venous blood samples (which have been filtered through organs and tissues) and arterial blood samples [taken before that filtration; (187)]. Additionally, there is a difference between blood samples taken from an indwelling cannula and a single needle stick. An indwelling cannula causes an immune response that can alter local cytokine production, and thus the resulting cytokine measurements may reflect local artifact rather than systemic change in concentrations (188). These are important factors to consider when designing and interpreting cytokine studies.

#### Plasma, Serum, and PBMC Sample Matrices Cannot Be Compared

When using peripheral blood samples, assays can be conducted on different sample matrices: whole blood, plasma, peripheral blood mononuclear cell (PBMC) isolate, or serum.

Whole blood can be:

- Collected into a tube with anticoagulants and then centrifuged. The resulting layers allow separation of **plasma** and of **PBMC**s.
- Collected into a tube without any additives and then centrifuged. After clotting factors are removed, the resulting liquid is **serum**.

During the processes required to make plasma or serum from blood, cells in the blood secrete inflammatory mediators that can alter cytokine measurements. For example, plasma preparation involves the removal of many proteins (e.g., fibrinogen), including the direct removal of circulating cytokines, obviously altering sample cytokine levels (189). During the coagulation process necessary for serum isolation, platelets release vascular endothelial growth factor, which can significantly alter cytokine levels (190). These are among the reasons that any attempt to compare cytokine levels across studies must take type of sample matrix into account.

Many other variables during sample handling and processing can affect cytokine levels in the sample matrix, including glass vs. plastic vials, type of anticoagulant (e.g., heparin, citrate, or EDTA), and centrifugation speed (190). Many studies in the ME/CFS cytokine literature differ in these details, limiting their comparability. Perhaps the most important methodological details involve time and temperature. Because both rapid degradation and *de novo* production of cytokines and other proteins occur inside of sample tubes (187, 191), without fast and careful processing, cytokine measurements may reflect processes that happened inside of a sample tube and not what happened in the bodies of study participants. While there is no way to completely avoid these confounds (192), these processes are greatly curtailed at −80°C but not at −20°C, meaning handling speed and storage temperature are crucial. The studies reviewed in Blundell et al. (104) ranged from immediate to 4 h between collection and plasma/serum separation, with many not reporting timing. Furthermore, 25 out of 57 studies in the ME/CFS cytokine literature either stored samples at −20°C or failed to report storage temperature at all (see Table A1). Thus, methodological details such as tubes, anticoagulants, centrifugation, and delays in processing are likely sources of type 2 error in the ME/CFS cytokine literature and limit the comparability across studies.

#### Bioassay, ELISA, and Multiplex Assay Results Cannot Be Compared, Even Across Kits of the Same Assay Type

After cytokine study samples have been collected, processed, and stored, they must be assayed. The assay methods for cytokine measurement have evolved over the past decades, and that evolution explains some current priorities in ME/CFS research. *Bioassays* are a form of assay that utilizes the biological activity of its target analyte to measure its concentration, while both enzyme-linked-immunosorbent assay (*ELISA*) and *multiplex* are immunoassays that usually use tagged antibodies. ELISA is the most commonly used method in ME/CFS (in the ME/CFS literature, all cytokine studies before 2007 were performed using bioassay or ELISA methods), but multiplex are becoming more common. ELISA formats are singleplex, meaning they characterize a single analyte (i.e., a single cytokine) while newer multiplex assays can measure many at the same time. Historically, ELISA is considered the gold standard because each kit can optimize sensitivity and specificity for the single specific cytokine being measured, and optimize for an expected concentration range (187).

Multiplex immunoassay methods are a more recent development, allowing for a larger number of cytokines (i.e., from 2 to 100+) to be characterized in the same assay. This is a seemingly-appealing option with the potential for identifying a putative cytokine profile in a complex multivariable disease, such as ME/CFS, that likely cannot be characterized by a single cytokine or other analyte. The ME/CFS literature has followed the advancing technology, generally shifting to multiplex. However, *multiplex sacrifices quality for quantity*. Because all cytokines are measured in the same multiplex kit well, there is inevitably cross-reactivity among the antibodies, and non-specificity with other non-cytokine proteins in the sample. Each manufacturer could theoretically optimize a select number of cytokines, but not all of them (e.g., the most sensitive and specific antibody for a given cytokine would have to be replaced by another antibody that is less cross-reactive). Companies also continuously develop new, revamped kits that cannot necessarily be compared to previous versions manufactured by the same company. In other words, one manufacturer's newest multiplex kit model may be particularly good at measuring IL-1β and bad at measuring TNF-α, while the inverse is true for that manufacturer's previous model, or another manufacturer's newest kit model. Furthermore, there can be a large range of concentrations among various cytokines in a given sample, and multiplex kits are unable to maximize sensitivity across that range. Therefore, a given kit may be relatively good at measuring high concentrations of IL-1β but lack sensitivity at lower levels. These forms of variance are true across the scores of cytokines each manufacturer advertises an ability to measure.

Currently, there are no standardized regulatory guidelines for the quality and validity of multiplex assays (193). Concordance between ELISA and multiplex varies widely and is especially poor if plasma or serum is used (189, 194); these are the most common sample types in ME/CFS, meaning ME/CFS studies using ELISA cannot be meaningfully compared to those using multiplex. Until multiplex methods are standardized, the best-case (but impractical) scenario for a researcher interested in 20 specific cytokines would be using 20 separate ELISA kits as opposed to using a 20-cytokine multiplex kit. However, absolute cytokine concentrations would not be comparable across studies if different researchers were to use kits from different manufacturers (195, 196). This is exactly what has happened in the ME/CFS literature, where many different kit manufacturers have been used (see Table A1). Cross-manufacturer differences in reported absolute values of cytokines occur because they are completely dependent on the standard curves from each kit, and studies have shown significant variation in standard curves across different manufacturers (195, 196). Taking all variables into account, it is unsurprising that *many studies have found profound differences in absolute cytokine levels across manufacturers and kits, even when compared on the same sample* (196–201). This clearly limits the ability for different studies in the ME/CFS cytokine literature to be compared.

Table A1 lists the various manufacturers and kit models used in the ME/CFS cytokine literature. Blundell et al. (104) correctly identified the importance of bioassay vs. immunoassay for a single cytokine (TGF-β), but this distinction was not made for any other cytokine. Furthermore, the distinction was not made between ELISA and multiplex immunoassays, nor was manufacturer or kit model taken into account for any cytokine. These details introduce enough variance as to make any attempted comparison of absolute cytokine concentrations in the ME/CFS literature indecipherable. A seemingly reasonable solution would be for all research groups to use the same assay kit model from the same manufacturer. However, we believe that peripheral cytokines are a fundamentally noisy variable and that this fact must be taken into account when considering the implications of any cytokine study.

#### The Same Exact Lab, Personnel, and Protocol Will Likely Get Different Results From the Same Manufacturer's Kit

**Figure 2:**
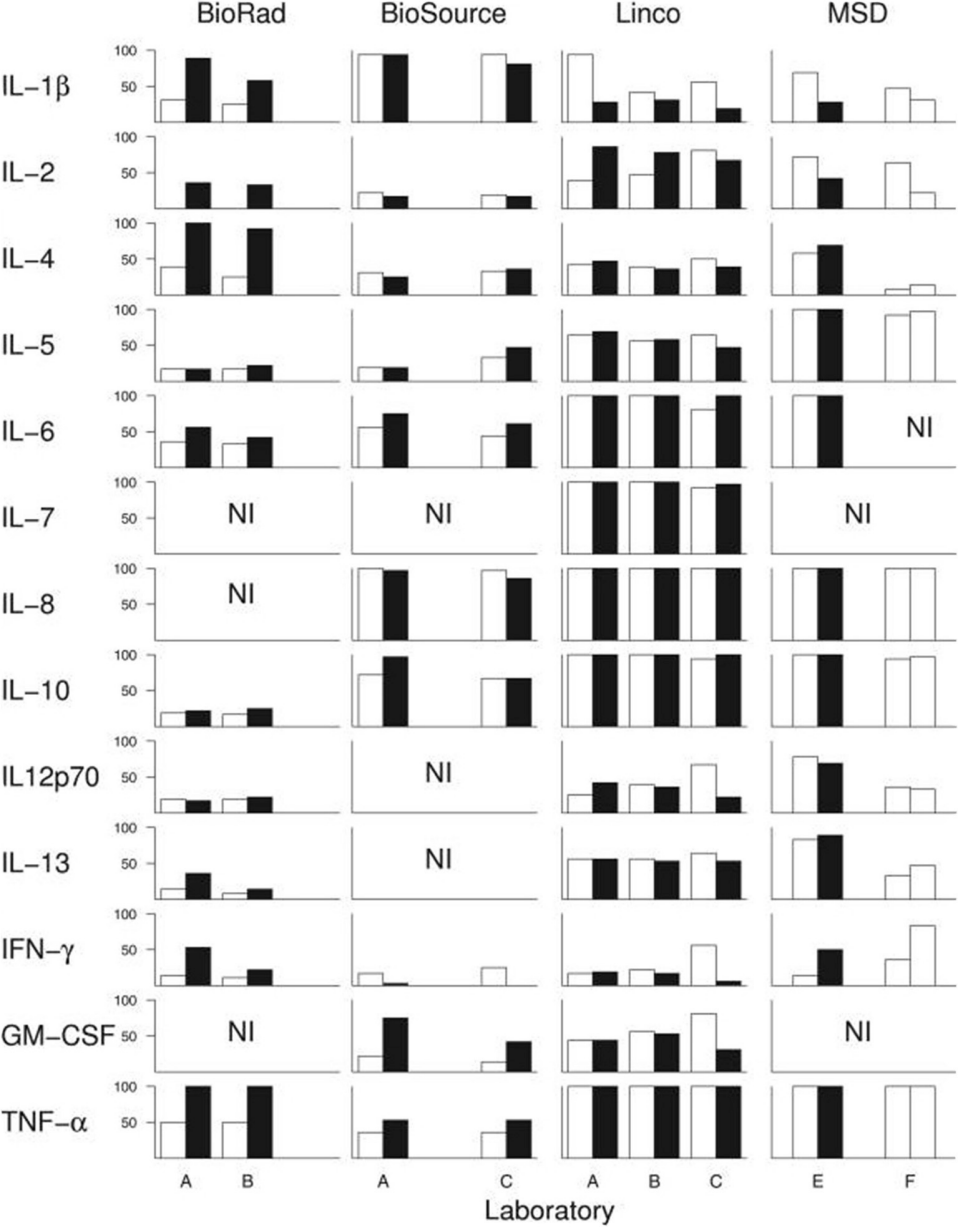
Breen et al. (198) conducted an experiment to test whether widely-used cytokine assays yield consistent results for 13 different cytokines. The same laboratories ran four different multiplex cytokine assay kits more than once on the same serum samples. Black and white bars represent assay kit data from different lots. Bars indicate percentage of serum samples (*n* = 36) with detectable levels of the indicated cytokine. A-F denote the six different labs in which the assays were conducted. NI: cytokines not included in each kit. Figure reproduced from Breen et al. (198). Reproduced with permission from American Society for Microbiology.

Assuming that there actually is a predictable, consistent peripheral “cytokine profile” in a complex illness such as ME/CFS, one potential solution to some of the above-described issues is if a single lab were to use the exact same techniques, equipment, and procedures across multiple studies, or if different labs standardized these procedures. However, empirical evidence shows that this is not the case. An experienced immunology lab, led by a PI with decades of experience and over 100 publications, conducted a within- and between-lab comparison study. Breen et al. (198) compared the ability of four multiplex kits to detect 13 cytokines in human plasma and serum. The four kits were tested on the same sample across six different laboratories and across multiple lots of the same kit. Their results showed a large amount of variance both within the same lab and across multiple labs. While all 13 cytokines were detected by at least one kit, none of the kits were able to detect all 13 cytokines. Additionally, their results alarmingly indicate that each cytokine within each multiplex kit had at least one significant lab and/or lot effect. In other words, *measuring the same sample twice with the same kit in the same laboratory following the same strict protocol yielded significant differences in absolute cytokine values* (Figure 2).

However, the results of the comparison demonstrated that while each of the kits varied in their sensitivity to detect the absolute concentration of cytokines, the kits detected similar cytokine patterns (relative concentrations, as opposed to absolute concentration). These findings contribute to our recommendation that cytokine assays are best suited to measuring relative changes in cytokine concentrations in a within-subject study design, rather than comparing absolute concentrations across groups (described below).

### Cytokines Can Be Highly Influenced by Individual Behavior

A final note of warning against overinterpreting studies of peripheral cytokines is that study participants can contribute noise in myriad ways. Factors that can significantly affect circulating cytokine levels within an individual include: time of day (202–204), status of alcohol, nicotine, or other drug use (205–211), quality and amount of sleep (212), acute and chronic stress (213), acute and chronic fitness habits specific to type of exercise (214–216), sex (217, 218), phase of menstrual cycle (219, 220), age (221), chronic dietary patterns (222), and acute differences immediately following a meal (223, 224). Thus, even eating a spicy burrito with extra guacamole the day of sample collection will result in a different cytokine profile than eating Indian food or a slice of chocolate cake. A research participant adding sour cream to the mashed potatoes they had for lunch will alter their cytokine profile. Capsaicin, the main source of heat in hot peppers, alters levels of IL-6, IL-10, TNFα, NOx, and MDA (225), and the natural sugars in avocado alter gene expression of IL-1α, IL-6, and IL-8 (227, 228). The bacteria used in dairy (i.e., the sour cream on the mashed potatoes) increase IL-1β, TNFα, and IFNγ (229, 230). Cumin, a spice commonly used in Indian cuisine, reduces expression of inflammatory cytokines CXL-1 and−2, TNFα, IL-1β, IL-6, and IL-18 (231, 232). Chocolate increases IL-10 and IL-1β (233). Clearly, cytokines can be affected by a huge number of variables unrelated to disease.

This type of variance, driven by individual behaviors, could be reasonably well explained in a single study using a within-subjects design. However, it can prevent comparability across studies that use different designs. For example, a study that collects blood samples during fasting cannot be compared to studies of non-fasting individuals undergoing exercise challenge. This type of variability in study design is widespread in the ME/CFS cytokine literature (see Table A1).

### Are Peripheral Circulating Cytokines Useful at All?

Given how cytokines work biologically, we do not believe that a consistent and stable proinflammatory circulating cytokine profile exists in patients with ME/CFS in comparison to controls, nor do we believe that finding such a profile is a realistic goal. Cytokines do not normally function as circulating endocrine molecules, and their presence in the periphery mostly represents spillover from their actual site of action. This biology also limits the value of any peripheral cytokine profile in elucidating the underlying pathophysiology of ME/CFS or any other chronic inflammatory condition. Cytokine measurement in the periphery is beset by innumerable confounds: biological, methodological, and behavioral. Detailed reporting of methods will help inform comparability across studies, while study designs with within-subjects measurements across multiple timepoints can help explain some of the behavioral variance.

We would argue that the most effective way to use peripheral cytokines in the characterization of ME/CFS patients is through within-subject or mixed-model challenge study designs (e.g., measuring before and after an exercise challenge, with BMI- and daily activity-matched controls). In such a study, cytokine levels would be most meaningful as a complementary measure, as opposed to a primary outcome measure. For example, cerebrospinal fluid could be sampled at both timepoints in a study measuring cognitive performance at baseline and during post-exercise symptom provocation. In such an example, if cognitive performance negatively correlates with a general increase in proinflammatory cytokines, this is indirect evidence that neuroinflammation is part of “brain fog.” This approach moves the focus of cytokine studies away from whether a distinct cytokine profile exists in ME/CFS patients, and toward the use of cytokines for understanding the mechanisms of key symptoms.

## Conclusion

The above review focused on neuroinflammation and the methods used to measure it. We argued for the importance of anchoring methodological details in known biological mechanisms and existing research literature.

The ME/CFS research field has been stuck in a somewhat defensive posture, with a focus on demonstrating “this is a real condition” by showing significant biological differences between patients and controls. We believe this has led to a situation in which too much is made of the specifics reported by descriptive studies (such as the average “cytokine profile” present in cases vs. controls at the moment of assay) and not enough emphasis has been placed on potential mechanisms driving symptoms. The field is ready to move past proving “this is a real condition” and to start elucidating the specific relationship of ME/CFS symptoms to neuroinflammation.

Moving past a defensive posture and toward understanding pathophysiology requires careful focus on research methods. In designing a study, a goal of ME/CFS researchers should be to determine if a significant result can actually inform disease mechanisms, or if it is simply a reportable difference between patients and controls. For example, a PET study of TSPO binding may find differences between patients and controls when using a cerebellum reference, and this holds some value for the “this is a real condition” argument. But because of the difficulty in interpretation, such a study is less valuable for discerning actual pathophysiology.

In consideration of neuroinflammation-related mechanisms and research methods, the following recommendations emerge:

- The relationship of ME/CFS to neuroinflammation is a fundamental question that needs to be directly addressed from multiple research angles.
- The existing neuroinflammation basic science literature should serve as a guide for choosing ROIs in ME/CFS brain scan studies.
- ME/CFS causes changes to patients' lives that could accidentally be explaining some study results (i.e., sedentary lifestyle or diet can affect cytokines). This makes careful selection of control groups particularly important.
- Cytokines seem attractive because they are easy to collect and measure, but are a very noisy variable and the specific findings of any given study should not be overinterpreted.
- Some methodological details are so fundamental (e.g., brainstem registration, or selection of a “baseline” reference brain region or metabolite, or choosing between blood serum and cerebrospinal fluid) that they can be completely responsible for a study's results or lack thereof.

## Author Contributions

All authors listed have made a substantial, direct and intellectual contribution to the work, and approved it for publication.

### Conflict of Interest

The authors declare that the research was conducted in the absence of any commercial or financial relationships that could be construed as a potential conflict of interest.

## Appendix

**Table A1:** Cytokine studies of ME/CFS.

		**Sample handling and processing**				**Assays**			**Assay results**		
**Study**	**Diagnostic criteria**	**Sample matrix**	**Collection (Specifications of note)**	**Time of collection**	**Storage**	**Method**	**Manufacturer**	**Kits**	**Increase**	**Decrease**	**No difference**
Lynn et al. 2018 (109)	CDC 1994	Serum	samples taken at 30min intervals on two consecutive days	10:00 a.m.	−80°C	Multiplex	BD Biosciences	Human CBA kit	IL-6, TNFα (response to low dose dex, LPS)	IP-10, IL-12/23p40in ME/CFS compared to healthy controls	
Richardson et al. 2018 (110)	CCC 2003	Serum	non-fasting blood samples collected after 20-min standing test	–	–	Both	BD Biosciences; activin ELISA supplied by Oxford Brookes University	Human CBA kit 560484	serum activin B		IL-2, IL-4, IL-6, IL-10, TNF, IFN γ, IL-17A, activin A
Oka et al. 2018 (111)	CDC 1994, ICC 2011, and SEID 2015 (112)	Serum and plasma (TGF-β1, BDNF)	after 8 weeks of intervention, blood sampling before and after the last session of interventional yoga	2:00–4:00.p.m	−80°C	ELISA	Fujirebio, R&D, pbl assay science, BioSource Europe S.A.; R&D	IL-6 CLEIA cartridge; Quantikine high-sensitivity ELISA human TNF-α immunoassay; VeriKine Human Interferon Alpha Multi-Subtype Serum ELISA kit; MEDGENIX human IFNγ EASIA kit; Quantikine ELISA human TGF-β1 kit, BDNF kit		TNF-α	IL-6; IFN-α, TGF-β1, BDNF; below level of detection: IFN-γ
Moneghetti et al. 2018 (113)	CDC 1994 and ICC 2011 for PEM	Serum	fasting blood sample	morning	−80°C	Multiplex	Affymetrix	51-Plex Luminex bead kit	Increased in ME/CFS but not healthy controls following exercise: CXCL 10	Decreased in ME/CFS but not healthy controls following exercise: IL-8, CCL4, TNF-β, ICAM-1	IL-1α, IL-1β, IL-1RA, IL-2, IL-4, IL-5, IL-6, IL-7, IL-10, IL12p40, IL12p70, IL-13, IL-15, IL-17, IL-17F, IL-18, LIF, FGF-β, HGF, NGF, PDGF-BB, TGFα, TGF-β1, VEGF, G-CSF, GM-CSF, M-CSF, SCF, CCL2, CCL3, CCL5, CCL7, CXCL1, CXCL5, CXCL9, CCL11, IFN-α, IFN-β, IFN-⋎, VCAM-1, CD40L, FASL, Leptin, PAI-1, Resistin, TNF-α, TRAIL
Wyller et al. 2017 (114)	CDC 1994 and CCC 2003	Plasma	fasting blood sample, no tobacco or caffeine	7:30–9:30 a.m.	−80°C	Multiplex	Bio-Rad Laboratories	Bio-Plex Human TGF-β 3-Plex			TGF-β1, TGF-β2, TGF-β3
Roerink et al. 2017 (115)	CDC 1994	Plasma	before and after 4 weeks of treatment	morning	−80°C	Multiplex, ELISA (TGF-β)	Olink Proteomics AB; R&D	Proseek Multiplex Inflammation panel; TGF-β duo-set DY240	Increased in ME/CFS compared to healthy controls at baseline: IL-12p40, CSF-1		CD40L, CCL2, IL-7, IL-8, CCL11, IL-6, IL-10, CXCL10, CXCL9, TRAIL, TNF-β, TGF-α, TGF-β1 Below level of detection: IFN-γ, IL-1α, IL-2, IL-4, IL17A, TNF
Milrad et al. 2018 (116)	CDC 1994	Plasma	–		−80°C	Multiplex	Quansys	Q-plex Human cytokine screen	Higher levels of cortisol predicted higher levels of IL-2, IL-6, TNF-α		
Montoya et al. 2017 (117)	CDC 1994	Serum	–	8:30 a.m.−3:30 p.m.	−80°C	Multiplex	Affymetrix	51-multiplex array	TGF-β; IL-13 in severe group (when stratified by severity); leptin in mild group; significant upward linear trend across severity: CCL11, CXCL1, CXCL10, G-CSF, GM-CSF, IFN-γ, IL-4, IL-5, IL-7, IL-12p70, IL-13, IL-17F, leptin, LIF, NGF, SCF, TGF-α	resistin in mild and severe groups; significant nonlinear inverted trend: ICAM1, resistin	CCL2, CCL3, CCL4, CCL5, CCL7, CD40L, CXCL5, CXCL9, FASL, FGF-basic, HGF, IFN-α, IFN-β, IL-1RA, 1L-1α, IL-1β, IL-2, IL-6, IL-8, IL-10, IL-12p40, IL-15, IL-17, M-CSF, PAI-1, PDGF-BB, TNF-a, TNF-β, TRAIL, VCAM1, VEGF
Nagy-Szakal et al. 2017 (118)	CDC 1994 and/or CCC 2003	Plasma	shipped from clinical sites	–	−80°C	Multiplex	Affymetrix	Customized Procarta immunoassay (61-plex)			IL1α, IL1β, IL1RA, IL18, IL2, IL4, IL7, IL9, IL13, IL15, IL5, IL6, LIF, IL31, IL10, IL21, IL22, IL12p40, IL12p70, IL23, IL27, IL17A, IL17F, IFNα2, IFNβ, IFNγ, TNFα (TNFSF2), TNFβ (TNFSF1), sFasL (TNFSF6), TRAIL (TNFSF10), CCL2 (MCP1), CCL3 (MIP1a), CCL4 (MIP1b), CCL5 (RANTES), CCL7 (MCP3), CCL11 (eotaxin), CXCL1 (GROa), CXCL8 (IL8), CXCL9 (MIG), CXCL10 (IP10), CXCL12a (SDF1a), PDGFBB, VEGFA, VEGFD, sICAM1 (CD54), VCAM1 (CD106), serpin E1 (PAI1), leptin, resistin, TGFα, TGFβ, FGFβ, βNGF, HGF, SCF, MCSF (CSF1), GMCSF(CSF2), GCSF (CSF3), PlGF1, EGF, BDNF
Hornig et al. 2017 (119)	CDC 1994 and/or CCC 2003	CSF	CSF samples from biobank	–	−80°C	Multiplex	Affymetrix	Customized Procarta immunoassay (51-plex)	FGFb in Classical-ME/CFS-short duration compared to Atypical-ME/CFS-short duration; SCF in Atypical-ME/CFS compared to Classical-ME/CFS irrespective of illness duration	IL1β, IL5, IL7, IL13, IL17A, IFNα2, IFNγ, TNFα, TRAIL (TNFSF10), CCL2, CCL7, CXCL5, CXCL9, CSF3 (GCSF), βNGF, resistin, serpin E1 (PAI1) in Atypical-ME/CFS-short duration compared to Classical-ME/CFS-short duration;IL7, IL17A, CXCL9, serpin E1 in Atypical-ME/CFS-short duration compared to Classical-ME/CFS-long duration;IL5, IL13, IL17A, CXCL9, in Atypical-ME/CFS-long duration compared to Classical-ME/CFS-short duration; IL6, IL17A in Atypical-ME/CFS-long duration compared to Classical-ME/CFS-long duration	IL1ra, IL1α, IL2, IL4, CXCL8 (IL8), IL10, IL12p40, IL12p70, IL15, IL17F, TNFβ, CD40L, sFasL, CCL3 (MIP1α), CCL4 (MIP1β), CCL5 (RANTES), CCL11 (eotaxin), CXCL1 (GROα), CXCL10 (IP10), TGFα, TGFβ, CSF1 (MCSF), CSF2 (GMCSF), PDGFBB, HGF, VEGFA, LIF, leptin, sICAM1 (CD54), VCAM1 (CD106)
Hanevik et al. 2017 (120)	CDC 1994	Stimulated PBMC culture	fasting blood samples	8:00–9:00 a.m.	−80°C	Multiplex	Bio-Rad Laboratories	Bioplex assays, kits not specified			IFN-γ, TNF-α, IL-1β, IL-2, IL-4, IL-6, IL-9, IL-10, IL-13, IL-17A, IL-22, MIP-1α, MIP-1β, TGFβ1, TGFβ2, TGFβ3, GM-CSF
Lidbury et al. 2017 (121)	CCC 2003	Serum	non-fasting samples collected after 20-min standing test	–	–	Both	BD Biosciences; activin kit supplied by Oxford Brookes University	Human CBA kit 560484	Activin B		IL-2, IL-4, IL-6, IL-10, activin A, follistatin; below levels of detection: IL-17A, IFN-γ, TNF-α
Milrad et al. 2017 (122)	CDC 1994	Plasma	x	11:00 a.m.−3:00 p.m.	−80°C	Multiplex	Quansys Biosciences	Q-plex Human cytokine screen	IL-1β, IL-6, TNF-a within CFS patients associated with poor sleep quality		
Lunde et al. 2016 (123)	CDC 1994 and CCC 2003	Serum	x	–	−80°C	ELISA	R&D; Invitrogen/Life technologies	BAFF and APRIL kits	BAFF, APRIL (baseline relative to healthy controls); BAFF, APRIL (post-intervention relative to baseline)		
Huth et al. 2016 (124)	CDC 1994	PBMC	x	7:30–10:00 a.m.	–	Neither	BD Biosciences; Biolegend	intracellular staining of stimulated and unstimulated PBMC cultures			IFN-γ, TNF-α and GM-CSF increased in culture after challenge, but no difference between groups
Russell et al. 2016 (125)	Jason revision for pediatrics of CDC 1994 and ICD, Reeves et al. 2005 (126)	Plasma	fasting blood sample	morning	−80°C	Multiplex	Quansys Biosciences	Q-plex Human cytokine screen (16-plex)	IL-4, IL-5, IL-12, LTα in 50+ y.o. ME/CFS females relative to healthy controls; IL-8 in ME/CFS adolescent females relative to healthy controls	IL-8, IL-15 in 50+ y.o. ME/CFS females relative to healthy controls; IL-23 in ME/CFS adolescent females relative to healthy controls	IL-1α, IL-1β, IL-2, IL-6, IL-10, IL-13, IL-17, IFNγ, TNFα
Landi et al. 2016 (127)	CDC 1994 and/or CCC 2003	Plasma	samples from Solve ME/CFS BioBank	–	−80°C	Multiplex	Meso Scale Discovery	MSD Human V-PLEX Plus Kits: Chemokine Panel 1, Cytokine Panel 1, and Pro-inflammatory Panel 1; Human Eotaxin-2 Kit, a custom-designed 3-Plex kit, a custom-designed 1-Plex kit	CCL24 univariate analysis	IL-16, IL-7, VEGF-A, CXCL9, CX3CL1 univariate analysis; IL-16, IL-7, VEGF-A by multivariate cluster analysis	IL-17A, TNFβ, CCL19, CCL11, IL-1β, TNFα, CCL3, CCL17, CCL2, IFN-g, IL-15, CCL26, IL-6, IL-12/23p40, CCL22, IL-5, CCL13, IL-1α, CCL4, GM-CSF, IL-10, IL-4, IL-13, IL-2, CXCL10, IL-12p70, IL-8, B2M
Hardcastle et al. 2015 (128)	CDC 1994	Serum	non-fasting blood sample	8:30–11:30 a.m.	–	Multiplex	BioRad	BioPlex Pro human cytokine 27-plex	IL-1β in moderate compared to severe ME/CFS; RANTES in moderate compared to severe ME/CFS and healthy controls; IFN-γ in severe compared to moderate ME/CFS; IL-7, IL-8 in severe compared to moderate ME/CFS and healthy controls	IL-6 in moderate compared to severe ME/CFS and healthy controls	IL-1ra, IL-2, IL-4, IL-5, IL-6, IL-9, IL-10, IL-12p70, IL-13, IL-17, FGF, eotaxin, G-CSF, GM-CSF, IP-10, PDGF-BB, TNF-α, VEGF, MCP1, MIP1a, and MIP1b
Peterson et al. 2015 (129)	CDC 1994	CSF	CSF samples via lumbar puncture	–	−80°C	Multiplex	BioRad	BioPlex Pro human cytokine 27-plex		IL-10	IL-1rα, IL-2, IL-6, IL-7, IL-8, IL-9, IL-12p70, IL-13, IL-15, IL-17, basic FGF, eotaxin, G-CSF, GM-CSF, IFN-γ, IP-10, MCP-1, RANTES, TNF-α, and PDGF-BB Below limit of detection: IL-1β, IL-4, MIP-1α, MIP-1β, IL-5 (VEGF listed in multiplex section)
Khaiboullina et al. 2015 (130)	CDC 1994 or Carruthers et al. 2011, 2003 (131, 132)	Serum	some blood samples shipped overnight	–	−80°C	Multiplex	Bio-Rad Laboratories	Bio-Plex Human Cytokine 27-Plex Panel, Bio-Plex Pro Human Chemokine Panels (40-plex), Bio-Plex Pro Human Th17 Cytokine Panels, Bio-Plex Cytokine 21-Plex Panels	CCL1, CCL2, CCL20, CCL3, CXCL10, IFN-γ, IL-1, IL-10, IL13, IL-1β, IL25, IL-31, IL-4, IL-6, IL-7, IL12 (p75), TNF-α	CCL11, CCL17, CCL19, CCL21, CCL25, CCL26, CCL3, CCL4, CCL5, CCL8, CSF1, CSF3, CX3CL1, CXCL1, CXCL13, CXCL6, CXCL8, HGF, IL-17F, IL5, IL-9, LIF, MIF, PDGF, TRAIL, VEGF	CCL13, CCL22, CCL23, CCL24, CCL27, CCL7, CXCL11, CXCL12a, CXCL12ab, CXCL16, CXCL2, CXCL5, CXCL9, FGF, GMCSF, IFN-α, IL-12 (p40), IL-15, IL-16, IL17A, IL-18, IL-1RA, IL-1α, IL-2, IL-21, IL-22, IL-23, IL-3, IL-33, IL-2RA, sCD40L, SCF, SCGF-β, TNF-β, β-NGF
Wyller et al. 2015 (133)	Royal College of Pediatrics & Child Health (2004) (134); National Institutes of Health and Clinical Excellence (2007) (135)	Plasma	fasting blood samples; abstained from tobacco and caffeine for 48 h	7:30–9:30 a.m.	−80°C	Multiplex	Bio-Rad Laboratories	Bio-Plex Human Cytokine 27-Plex Panel			IL-1β, IL-1RA, IL-2, IL-4, IL-5, IL-6, IL-7. IL8, IL-9, IL-10, IL-12, IL-13, IL-17, IFN-γ, CCL2, CCL3, CCL4, CCL5, CXCL10, PDGF-BB, VEGF, FGF, TNF
Hornig et al. 2015 (136)	CDC 1994, CCC 2003, or Jason et al. (137)	Plasma	samples shipped overnight	10:00 a.m.−2:00 p.m.	−80°C	Multiplex	Affymetrix	customized Procarta immunoassay	Leptin	IL-6, IL-8, IL-10, LT-α, IL17A, sFasL, CXCL10, M-CSF	TGF-β, IL-1β, IL-1α, TNF-α, IFN-α2, IL-2, IL-12p40, IL-12p70, IFN-c, IL-4, IL-13, IL-5, IL-15, IL-7, GMCSF, LIF, CD40L, TRAIL, CCL2, CCL3, CCL4, CCL5, CCL7, CCL11, CXCL1, CXCL5, CXCL9, PDGF-BB, VEGFA, sICAM-1, VCAM-1, TGF-α, FGFb, bNGF, HGF, SCF, G-CSF, IL17F, IFN-B, serpin E1, resistin
Neu et al. 2014 (138)	CDC 1994	Serum	samples collected after 2nd night of polysomnography (indwelling cannula)	early morning	−20°C	Multiplex	BD Biosciences	CBA Human flex-set kit	IL-1β, TNF-α, IL8, IL-10	IL-6, IFN-γ	
Nakatomi et al. 2014 (8)	CDC 1994 and Carruthers	Serum	–	–	−80°C	–	analyzed by the Mitsubishi Chemical Mediance Corps	–			IL-1β, IL-6, TNF-α, IFN- γ
Garcia et al. 2014 (139)	CDC 1994	Serum	x	–	–	Multiplex	Millipore	–	IL-6, IL-2, IL12p70, IFN-c, GMCSF, CXCL10		IL-1β, IL-1α, TNF-α, IL-8, IL-10, IFN-γ, IL-4, LT-α, IL-5, IL-7, CCL2, CCL3, CCL4, IL-3
Nakamura et al. 2013 (140)	CDC 1994	Plasma	venous sampling throughout 3 nights of different sleep conditions: normal, after exercise testing, and without sleep (indwelling cannula)	1 h after cannula placement (awake), 1:00, 3:00, 5:00, between 7:15 a.m.−8:00 a.m. after waking	−80°C	Multiplex	Millipore	Milliplex human multicytokine detection system			IL-1β, IL-6, TNF-α, IL-8, IL-10, IL-4
Maes et al. 2013 (141)	CDC 1994	Plasma	fasting blood samples	8:30–11:30 a.m.	–	ELISA	R&D, GE Healthcare UK Ltd.	Quantikine Human TNF-α Immunoassay; Amersham Interleukin-1 alpha [(h) IL-1α]; Amersham Interleukin-1 beta [(h) IL-1β]	IL-1β, IL-1α, TNF-α		
Stringer et al. 2013 (142)	CDC 1994	Serum	25 consecutive days of blood draws, site of blood draw rotated regularly	Visits were held within a 2 h window	−80°C	Multiplex	Affymetrix	Human 51-plex Luminex	Leptin predicted daily fatigue severity in ME/CFS patients but not in healthy controls		ICAM1, TGF-b, IL10, PDGFBB, MCP3, IL1B, ENA78, MCSF, IFN-a, IL12P40, FGF-b, RANTES, TNF-b, IL1RA, PAI1, FASL, VCAM1, MCP1, IP10, HGF, CD40L, IFN-g, IL2, VEGF, GMCSF, MIP1B, SCF, TNF-a, TRAIL, IL5, MIP1A, MIG, GCSF, RESISTIN, IL7, EOTAXIN, TGF-a, IL17F, IL15, IFN-g, IL13, IFN-b, IL12P70, IL6, IL4, NGF, IL1A, LIF, IL8, GRO-a
Lattie et al. 2012 (143)	CDC 1994	Plasma	x	11:00 a.m.−3:00 p.m.	−80°C	ELISA & Multiplex	Quansys Biosciences and R&D	Q-Plex Human Cytokine Screen; assayed in duplicate with R&D standard	IL-1β, IL-6		TNF-α, IL-10, IL-2
Smylie et al. 2013 (144)	CDC 1994 and Reeves et al. 2005 (126)	Plasma	blood drawn 3x during exercise challenge: at rest (T0), at VO_2_ max (T1), 4 h after exercise (T3)	–	−80°C	Multiplex	Quansys Biosciences	Q-Plex Human Cytokine Screen (16-plex)	Males: IL-2 (T0, T1, T2), IL23 (T0, T2); Females: IL-1α (T1)	Females: IL-4 (T1)	Females: IL-1b, IL-6, TNF-α, IL-8, IL-10, IL-2, IL-12, IFN-c, IL-13, TNF-β, IL-5, IL-23, IL-17, IL-15; Males: IL-1b, IL-1α, IL-6, TNF, IL-8, IL-10, IL-12, IFN-c, IL-4, IL-13, TNF-β, IL-5, IL-17, IL-15
Broderick et al. 2012 (145)	Reeves et al. 2005 (126) and Jason et al. 2006 (146)	Plasma	fasting blood samples	morning	−80°C	Multiplex	Quansys Biosciences	Q-Plex Human Cytokine Screen (16-plex)	IL-8	IL-23	IL-1β, IL-1α, IL-6, TNF-α, IL-10, IFN-α, IL-2, IL-12p70, IL-4, IL-13, TNF-β, IL-5, IL-17, IL-15
Maes et al. 2012 (147)	CDC 1994	Plasma	fasting blood samples	8:30–11:30 a.m.	–	ELISA	R&D, GE Healthcare UK Ltd.	Quantikine Human TNF-α Immunoassay; Amersham Interleukin-1 alpha [(h) IL-1α]; Amersham Interleukin-1 beta [(h) IL-1β]	IL-1β, IL-1α, TNF-α		
Nas et al. 2011 (148)	ICC 2011	Serum	–	–	–	–	DPC Immulite 1000 Chemistry Analyzer	IMMULITE 1000 analyzers, kits not specified	IL-6, IL2r		IL-8
White et al. 2010 (149)	CDC 1994	Serum	blood samples at baseline, 0.5, 8, 24, and 48 h post exercise	–	−80°C	Multiplex	Developed at the ARUP Institute for Clinical and Experimental Research (Salt Lake City, UT)	–		CD40L	IL-1β, IL-6, TNF-α, IL-8, IL-10, IL-2, IL-12, IFN-γ, IL-4, IL-13
Nakamura et al. 2010 (150)	CDC 1994	Plasma	venous sampling throughout the night while asleep (indwelling cannula)	1:00, 3:00, 5:00, 7-7:30 a.m.	−80°C	Multiplex	Millipore	Beadlyte human multicytokine detection system 2	IL-10 in CFS without FM compared to controls at 3:00am and 5:00am, and compared to CFS with FM at 5:00am		IL-1β, IL-6, TNF, IL-8, IL-4
Nijs et al. 2010 (151)	CDC 1994	–	blood samples taken before and 1 h after exercise	–	–	ELISA	Amersham Biosciences Europe GmbH, Pierce Biotechnology Inc.	Biotrak Easy ELISA RPN5971, Endogen Human IL-1β ELISA kit			Below level of detection: IL-1β
Robinson et al. 2010 (152)	CDC 1994	Plasma	blood sampled at rest, at point of exhaustion, and 24 h post exercise (indwelling cannula); after overnight fast and abstaining from alcohol, caffeine, and strenuous activity for 24 h	–	−80°C	ELISA	BD Biosciences	OptEIA			IL-6
Scully et al. 2010 (153)	CDC 1994	Plasma	x	–	−80°C	Multiplex	Meso Scale Discovery	–	IL-1β, IL-6, IL-8		TNF-α, IL-10, IFN-γ, IL-12p70, IL-13
Fletcher et al. 2009 (154)	CDC 1994, Reeves et al. 2005 (126)	Plasma	x	morning	−80°C	Multiplex	Quansys Biosciences	Q-Plex Human Cytokine-Screen (16-plex)	IL-1β, IL-1α, IL-6, IL-12, IL-4, IL-5, TNF-β	IL-8, IL-15, IL-13	TNF-α, IL-10, IL-2, IFN-γ, IL-13, IL-23, IL-17
Jammes et al. 2009 (155)	CDC 1994	Plasma	sampling throughout exercise protocol (indwelling cannula)	–	–	ELISA	R&D	Quantikine HS Human IL-6 Immunoassay D6050; Quantikine HS Human TNF-α DTA00C		Depressed IL-6 and TNF-α response to exercise in CFS (absence of significant post-exercise increase as in controls)	Baseline IL-6 and TNF-α
Nater et al. 2008 (156)	CDC 1994	Plasma	fasting blood samples taken 30 minutes after indwelling cannula was placed	7:30 a.m.	−80°C	ELISA	R&D	Quantikine HS Human IL-6 Immunoassay	lL-6 (not significant after controlling for BMI, even though BMI did not differ across groups)		
Spence et al. 2008 (157)	CDC 1994	Serum	x	same time of day	−70°C	ELISA	R&D	–			IL-1, TNF-α
Vollmer-Conna et al. 2007 (158)	CDC 1994	Serum	blood samples taken 1, 2, 3, 6, 12 months after infection onset	–	−80°C	Multiplex	BioRad	Bioplex			IL-1β, IL-2, IL-4, IL-6, IL-10, IL-12, TNF-α, IFN-γ; serum cytokine levels almost exclusively below detection level
Kennedy et al. 2004 (159)	CDC 1994	Platelet poor plasma	x	same time of day	–	ELISA	R&D	–	TGF-β1		
White et al. 2004 (160)	CDC 1994	Plasma	blood collected at baseline, immediately pre- & post-exercise, 3 h and 3 days post-exercise	9:30 a.m.−12:30 p.m.	–	ELISA	R&D	–	TGF-β1 at all time points compared to healthy controls; TNF-α at 3 h and 3 days post-exercise compared to healthy controls		IL-1-α
Visser et al. 2001 (161)	CDC 1994	WBC	fasting blood samples	7:00 a.m.−10:00 a.m.	−20°C	ELISA	Pharmingen, R&D, Biorad	method from Cheney et al. 1989 (162)			TNFα, IL-10, IL-12, IFN-c
Cannon et al. 1999 (163)	CDC 1988	Plasma	collected 24 h post exercise	9:00 a.m.	–	ELISA	R&D	–			IL-6 (majority of samples below detection level)
Buchwald et al. 1997 (164)	CDC 1988 and 1994	Serum	x	–	–	ELISA	Genzyme Diagnostics	Predicta			IL-6
Bennett et al. 1997 (165)	CDC 1988	Serum	samples shipped on dry ice 1 year before analysis	–	−20°C	Bioassay	R&D (IL-4-dependent HT-2 cell proliferation bioassay)	–	TGF-β		
MacDonald et al. 1996 (166)	CDC 1988	Serum	x	7:00–10:00 a.m.	–	ELISA	CCC (167)	For TGFβ: specially developed in a co-investigators lab; others not specified			TGF-β, IL-1β, IL-6, TNF-α
Swanink et al. 1996 (168)	Sharpe et al. 1991 (169)	Plasma, Serum (TGFβ)	no caffeine for 48 h; stopped all medications prior to blood draw	8:30–11:00 a.m.	–	ELISA	R&D, Endogen	Measured as previously described in Drenth et al. 1995 (170); Quantikine (TGFβ)			TGF-β, IL-1β, IL-1α, TNF
Peterson et al. 1994 (171)	CDC 1988 and Schluederberg et al. 1992 (172)	Serum	blood collected at rest, immediately after exercise, and 40 min after exercise	–	−70°C	ELISA, bioassay (TGFβ)	R&D (ELISA and IL-4-dependent HT-2 cell proliferation bioassay)	Measured as previously described in Chao et al. 1991 (167)	TGF-β elevated in CFS compared to healthy controls at rest and 40 min after exercise		Below limit of detection at all time points in CFS and healthy controls: IL-1β, IL-6, TNFα
Patarca et al. 1994 (173)	CDC 1988	Plasma and serum	once a month for 3 months	7:30–10:30 a.m.	−20°C	ELISA	Endogen, R&D, Amersham, Genzyme, T-Cell Diagnostics	Intertest-4, Biokine	TNF-α, TNF-β		IL-1β, IL-1α, IL-6, IL-2, IL-4, GMCSF
Lloyd et al. 1994 (174)	Lloyd 1988 and Holmes 1988	Serum	blood was collected prior to, during, 15 min after, 4, 24 h post exercise (indwelling cannula)	–	−70°C	ELISA, radioimmunoassay	Sucrosep; Centocor; Cistron Biotechnology; T Cell Sciences	Biokine TNF			IL-1β, TNF-α, IFN-α, IFN-γ: all cytokines at all time points were at or below the level of detection; no significant differences between CFS and healthy controls
Linde et al. 1992 (175)	CDC 1988	Serum	blood samples drawn >1 year after onset of symptoms & during a period of severe clinical symptoms (samples stored at room temperature for 2 h)	–	–	ELISA, radioimmunoassay, dissociation-enhanced lanthanide fluoroimmunoassay, EASIA	Delfia; RIA; Medgenix; Quantikine R&D	–	In CFS patients and acute infectious mononucleosis patients compared to controls: IL-1α		IL-1β, IL-6; IFN-γ (no difference between CFS patients and controls, but increased in acute infectious mononucleosis patients compared to controls); IFN-α: many samples below level of detection
Chao et al. 1991 (167)	CDC 1988	Serum	1x a day, 5 consecutive days; no anti-inflammatory drugs for at least 3 days prior to blood sampling	8:00–9:00 a.m.	−20°C	ELISA, bioassay (TGFβ)	R&D (ELISA and IL-4-dependent HT-2 cell proliferation bioassay)	–	TGF-β		IL-1β, IL-6, TNF: most samples had levels below detection limit and did not differ significantly between groups; IL-2, IL-4: levels below detection limit
Straus et al. 1989 (176)	CDC 1988	Serum, plasma (TNF)	samples shipped on dry ice	–	−20°C	ELISA, radioimmunoassay	–	sent to same laboratory as in Cheney et al. 1989 (162), no specifications			IL-1β, IFN-α, IFN-γ: most samples had levels below detection limit, no significant difference between groups; TNF, IL-2
Cheney et al. 1989 (162)	CDC 1988	Serum	samples were shipped from geographically separate regions	–	–	ELISA	Genzyme	sent to Specialty Laboratories, LA, no specifications	IL-2		

*Research articles compared in this table include the studies reviewed by Blundell et al. (104), as well as studies published since then (distinguished by the horizontal double line in the table). The newer studies were found by searching for “cytokine” along with “myalgic encephalomyelitis,” “chronic fatigue syndrome,” “myalgic encephalomyelitis/chronic fatigue syndrome,” relevant abbreviations, or a combination of the abbreviations. Studies were selected if they included an ME/CFS group and used a cytokine assay. Though not a complete systematic literature review, this table is intended to show the variability in methods (from sample collection and storage to assay selection) and reported results across ME/CFS cytokine studies. We use the terminology used by each paper, and report analytes that each paper defined as a cytokine. The table primarily addresses case-control studies so that methods could be compared. The table lists absolute values and does not include descriptions of multiple cytokines added to regression models. –, not specified/reported. x, no specifications of note for sample collection*.
