# Corrigendum: Neuroinflammation and Cytokines in Myalgic Encephalomyelitis/Chronic Fatigue Syndrome (ME/CFS): A Critical Review of Research Methods

**DOI:** 10.3389/fneur.2019.00316

**Published:** 2019-04-02

**Authors:** Michael B. VanElzakker, Sydney A. Brumfield, Paula S. Lara Mejia

**Affiliations:** Division of Neurotherapeutics, Massachusetts General Hospital, Harvard Medical School, Boston, MA, United States

**Keywords:** myalgic encephalomyelitis, neuroimaging, glia, microglia, PBR28, cytokines, translocator protein, positron emission tomography

In the original article, there was a mistake in Appendix Table A1, Cytokine studies of ME/CFS as published. In the “Montoya et al. (2017)” row, the words “and plasma” should have been removed from the “Sample matrix” column as only the serum was analyzed. The words “kit not specified” from the “Kits” column should also be removed. The specific model/catalog number of their 51-multiplex array was not specified, but the table's wording could be misinterpreted because Montoya et al. (2017) specified other assay details.

**Table A1:** Cytokine studies of ME/CFS.

**Study**	**Diagnostic criteria**	**Sample handling and processing**				**Assays**			**Assay results**		
		**Sample matrix**	**Collection (Specifications of note)**	**Time of collection**	**Storage**	**Method**	**Manufacturer**	**Kits**	**Increase**	**Decrease**	**No difference**
Lynn et al. 2018 (109)	Fukuda et al. 1994 (71)	Plasma	samples taken at 30 min intervals on two consecutive days	10:00 a.m.−12:00 p.m.	−80°C	Multiplex	BD Biosciences	Human CBA kit	IL-6, TNFα (response to low dose dex, LPS)	IP-10, IL-12/23p40	
Richardson et al. 2018 (110)	Carruthers et al. 2003 (223)	Serum	non-fasting blood samples collected after 20-min standing test	–	–	Both	BD Biosciences; activin ELISA supplied by Oxford Brookes University	Human CBA kit 560484	serum activin B		IL-2, IL-4, IL-6, IL-10, TNF, IFN γ, IL-17A, activin A
Oka et al. 2018 (111)	Fukuda et al. 1994 (71), Carruthers et al. 2011 (225), and SEID, 2015 (230)	Serum and plasma (TGF-β1, BDNF)	after 8 weeks of intervention, blood sampling before and after the last session	2:00–4:00 p.m.	−80°C	ELISA	Fujirebio, R&D, pbl assay science, BioSource Europe S.A.; R&D	IL-6 CLEIA cartridge; Quantikine high-sensitivity ELISA human TNF-α immunoassay; VeriKine Human Interferon Alpha Multi-Subtype Serum ELISA kit; MEDGENIX human IFNγ EASIA kit; Quantikine; Quantikine ELISA human TGF-β1 kit, BDNF kit		TNF-α	IL-6; IFN-α, IFN-γ; TGF-β1, BDNF
Moneghetti et al. 2018 (112)	Fukuda et al. 1994 (71) and Carruthers et al. 2011 (225) for PEM	Serum	fasting blood sample	morning	−80°C	Multiplex	Affymetrix	51-Plex Luminex bead kit	CXCL 10	IL-8, CXCL10, CCL4, TNF-β, ICAM-1	IL-1α, IL-1β, IL-1RA, IL-2, IL-4, IL-5, IL-6, IL-7, IL-8, IL-10, IL12p40, IL12p70, IL-13, IL-15, IL-17, IL-17F, IL-18, LIF
Milrad et al. 2018 (115)	Fukuda et al. 1994 (71)	Plasma	–	11:00 a.m.−3:00 p.m.	−80°C	Multiplex	Quansys (R&D)	Q-plex Human cytokine screen	IL-2, IL-6, TNF-α		
Wyller et al. 2017 (113)	Fukuda et al. 1994 (71) and Carruthers et al. 2003 (223)	Plasma	fasting blood sample, no tobacco	7:30–9:30 a.m.	−80°C	Multiplex	Bio-Rad Laboratories	Bio-Plex Human TGF-β 3-Plex			TGF-β1, TGF-β2, TGF-β3
Roerink et al. 2017 (114)	Fukuda et al. 1994 (71) and Carruthers et al. 2003 (223)	Plasma	before and after 4 weeks of treatment	–	−80°C	Multiplex	Olink Proteomics AB; R&D	Proseek Multiplex Inflammation panel; TGF-β duo-set DY240	Positively associated with risk of being an ME/CFS patient: CCL4, 1L12β, CCL11, FGF5, IL6, CCL23, CX3CL1, IL10, CXCL5	Negatively associated with risk of being an ME/CFS patient: CXCL6, CXCL10, CXCL9, CCL28, CCL25, CCL20, CCL19, TRAIL, TNFβ, FGF23	IFN-γ, IL-1α, IL-2, IL-4, IL17A, TNF, MCP-3, IL-17c, TSLP, PD-L1, IL-24, IL-13, IL-20, IL-33, LIF, IL-5
Montoya et al. 2017 (116)	Fukuda et al. 1994 (71)	Serum	–	8:30 a.m.−3:30 p.m.	−80°C	Multiplex	Affymetrix	51-multiplex array	TGF-β; IL-13 in severe group (when stratified by severity); significant upward linear trend across severity: CCL11, CXCL1, CXCL10, G-CSF, GM-CSF, IFN-γ, IL-4, IL-5, IL-7, IL-12p70, IL-13, IL-17F, leptin, LIF, NGF, SCF, TGF-α	resistin; significant nonlinear inverted trend: ICAM1, resistin	34 others from 51-multiplex array
Nagy-Szakal et al. 2017 (117)	Fukuda et al. 1994 (71) and Carruthers et al. 2003 (223)	Plasma	–	–	−80°C	Multiplex	Affymetrix	Customized Procarta immunoassay (61-plex)			IL1α, IL1β, IL1RA, IL18, IL2, IL4, IL7, IL9, IL13, IL15, IL5, IL6, LIF, IL31, IL10, IL21, IL22, IL12p40, IL12p70, IL23, IL27, IL17A, IL17F, IFNα2, IFNβ, IFNγ, TNFα (TNFSF2), TNFβ (TNFSF1), sFasL (TNFSF6), TRAIL (TNFSF10), CCL2 (MCP1), CCL3 (MIP1a), CCL4 (MIP1b), CCL5 (RANTES), CCL7 (MCP3), CCL11 (eotaxin), CXCL1 (GROa), CXCL8 (IL8), CXCL9 (MIG), CXCL10 (IP10), CXCL12a (SDF1a), PDGFBB, VEGFA, VEGFD, sICAM1 (CD54), VCAM1 (CD106), serpin E1 (PAI1), leptin, resistin, TGFα, TGFβ, FGFβ, βNGF, HGF, SCF, MCSF (CSF1), GMCSF(CSF2), GCSF (CSF3), PlGF1, EGF, BDNF
Hornig et al. 2017 (118)	Fukuda et al. 1994 (71) and /or Carruthers et al. 2003 (223)	CSF	CSF samples from biobank	–	−80°C	Multiplex	Affymetrix	Customized Procarta immunoassay (51-plex)	FGFb in Classical-ME/CFS-short duration compared to Atypical-ME/CFS-short duration; SCF in Atypical-ME/CFS compared to Classical-ME/CFS irrespective of illness duration	IL1β, IL5, IL7, IL13, IL17A, IFNα2, IFNγ, TNFα, TRAIL (TNFSF10), CCL2, CCL7, CXCL5, CXCL9, CSF3 (GCSF), βNGF, resistin, serpin E1 (PAI1) in Atypical-ME/CFS-short duration compared to Classical-ME/CFS-short duration; IL7, IL17, CXCL9, serpin E1 in Atypical-ME/CFS-short duration compared to Classical-ME/CFS-long duration; IL5, IL13, IL17, CXCL9, in Atypical-ME/CFS-long duration compared to Classical-ME/CFS-short duration; IL6, IL17 in Atypical-ME/CFS-long duration compared to Classical-ME/CFS-long duration	IL1ra, IL1α, IL2, IL4, CXCL8 (IL8), IL10, IL12p40, IL12p70, IL15, IL17F, TNFβ, CD40L, sFasL, CCL3 (MIP1α), CCL4 (MIP1β), CCL5 (RANTES), CCL11 (eotaxin), CXCL1 (GROα), CXCL10 (IP10), TGFα, TGFβ, CSF1 (MCSF), CSF2 (GMCSF), PDGFBB, HGF, VEGFA, LIF, leptin, sICAM1 (CD54), VCAM1 (CD106)
Hanevik et al. 2017 (119)	Fukuda et al. 1994 (71)	PBMC	fasting blood samples	8:00–9:00 a.m.	−80°C	Multiplex	Bio-Rad Laboratories	Bioplex assays, kits not specified	sCD40L in Giardia-exposed vs. unexposed ME/CFS subjects, and in post-infective-ME/CFS vs. no post-infective fatigue group		IFN-γ, TNF-α, IL-1β, IL-2, IL-4, IL-6, IL-9, IL-10, IL-13, IL-17A, IL-22, MIP-1α, MIP-1β, TGFβ1, TGFβ2, TGFβ3, GM-CSF
Lidbury et al. 2017 (120)	Carruthers et al. 2003 (223)	Serum	non-fasting samples collected after 20-min standing test	–	–	Both	BD Biosciences; activin kit supplied by Oxford Brookes University	Human CBA kit 560484	Activin B		IL-2, IL-4, IL-6, IL-10, IL-17A, TNF, IFN-α, activin A, follistatin
Milrad et al. 2017 (121)	Fukuda et al. 1994 (71)	Plasma	x	11:00 a.m.−3:00 p.m.	−80°C	ELISA	Quansys Biosciences	Q-plex Human cytokine screen	IL-1β, IL-6, TNF-a within CFS patients associated with poor sleep quality in ME/CFS		
Lunde et al. 2016 (122)	Fukuda et al. 1994 (71) and Carruthers et al. 2003 (223)	Serum and plasma	x	–	−80°C	ELISA	R&D; Invitrogen/Life technologies	BAFF and APRIL kits	BAFF in intervention group relative to baseline		APRIL
Huth et al. 2016 (123)	Fukuda et al. 1994 (71)	PBMC	x	7:30–10:00 a.m.	–	Neither	BD Biosciences; Biolegend	intracellular staining of stimulated and unstimulated PBMC cultures			IFN-γ, TNF-α and GM-CSF increased in culture after challenge, but no difference between groups
Russell et al. 2016 (124)	Jason et al. 2006 (226) and Fukuda et al. 1994 (71) and ICD	Plasma	fasting blood sample	morning	−80°C	ELISA	Quansys Biosciences	Q-plex Human cytokine screen (16-plex)	IL-4, IL-5, IL-12, LTα in ME/CFS patients relative to healthy controls; IL-8 in ME/CFS adolescents	IL-8, IL-15 ME/CFS patients relative to healthy controls; IL-23 in ME/CFS adolescents	IL-1α, IL-1β, IL-2, IL-6, IL-10, IL-13, IL-17, IFNγ, TNFα
Landi et al. 2016 (125)	Fukuda et al. 1994 (71) and Carruthers et al. 2003 (223)	Plasma	samples from Solve ME/CFS BioBank	–	−80°C	ELISA	Meso Scale Discovery	MSD Human V-PLEX Plus Kits: Chemokine Panel 1, Cytokine Panel 1, and Pro-inflammatory Panel 1; Human Eotaxin-2 Kit, a custom-designed 3-Plex kit, a custom-designed 1-Plex kit	CCL24 univariate analysis	IL-16, IL-7, VEGF-A, CXCL9, CX3CL1 univariate analysis; IL-16, IL-7, VEGF-A by multivariate cluster analysis	IL-17A, TNFβ, CCL19, CCL11, IL-1β, TNFα, CCL3, CCL17, CCL2, IFN-g, IL-15, CCL26, IL-6, IL-12/23p40, CCL22, IL-5, CCL13, IL-1α, CCL4, GM-CSF, IL-10, IL-4, IL-13, IL-2, CXCL10, IL-12p70, IL-8, B2M
Hardcastle et al. 2015 (126)	Fukuda et al. 1994 (71)	Serum	non-fasting blood sample	8:30–11:30 a.m.	–	Multiplex	BioRad	BioPlex Pro human cytokine	IL-1β in moderate compared to severe ME/CFS; IL-7, IL-8, RANTES in moderate compared to severe ME/CFS and healthy controls; IFN-γ in severe compared to moderate ME/CFS	IL-6 in moderate compared to severe ME/CFS and healthy controls,	IL-1ra, IL-2, IL-4, IL-5, IL-6, IL-9, IL-10, IL-12p70, IL-13, IL-17, FGF, eotaxin, G-CSF, GM-CSF, IP-10, PDGF-BB, TNF-α and VEGF
Peterson et al. 2015 (127)	Fukuda et al. 1994 (71)	CSF	CSF samples via lumbar puncture	–	−80°C	Multiplex	BioRad	BioPlex Pro human cytokine		IL-10	IL-1rα, IL-2, IL-6, IL-7, IL-8, IL-9, IL-12p70, IL-13, IL-15, IL-17, basic FGF, eotaxin, G-CSF, GM-CSF, IFN-γ, IP-10, MCP-1, RANTES, TNF-α, and PDGF-BB
Khaiboullina et al. 2015 (128)	Fukuda et al. 1994 (71) or ICC 2011	Serum	x	–	−80°C	Multiplex	Bio-Rad Laboratories	Bio-Plex Human Cytokine 27-Plex Panel	CCL1, CCL2, CCL20, CCL3, CXCL10, IFNc, IL-1, IL-10, IL13, IL-1β, IL25, IL-31, IL-4, IL-6, IL-7, IL12 (p75), TNF	CCL11, CCL17, CCL19, CCL21, CCL25, CCL26, CCL3, CCL4, CCL5, CCL8, CSF1, CSF3, CX3CL1, CXCL1, CXCL13, CXCL6, CXCL8, HGF, IL-17F, IL5, IL-9, LIF, MIF, PDGF, TRAIL, VEGF	CCL13, CCL22, CCL23, CCL24, CCL27, CCL7, CXCL11, CXCL12a, CXCL12ab, CXCL13, CXCL16, CXCL2, CXCL5, CXCL9, FGF, GMCSF, IFN-α, IL-12 (p40), IL-15, IL-16, IL17A, IL-18, IL-1RA, IL-1α, IL-1b, IL-2, IL-21, IL-22, IL-23, IL-3, IL-33, IL-6, IL-2RA, LIF, sCD40L, SCF, SCGF-b, TNF-β, b-NGF
Wyller et al. 2015 (129)	Fukuda et al. 1994 (71)	Plasma	fasting bood samples	7:30–9:30 a.m.	−80°C	Multiplex	Bio-Rad Laboratories	Bio-Plex Human Cytokine 27-Plex Panel			IL-1β, IL-1RA, IL-2, IL-4, IL-5, IL-6, IL-7. IL8, IL-9, IL-10, IL-12, IL-13, IL-17, IFN-c, CCL2, CCL3, CCL4, CCL5, CXCL10, PDGF-BB, VEGF, FGF, TNF
Hornig et al. 2015 (130)	Fukuda et al. 1994 (71) and Carruthers et al. 2003 (223)	Plasma	–	10:00 a.m.−2:00 p.m.	−80°C	ELISA	Affymetrix	customized Procarta immunoassay	Leptin	Il-6, IL-8, IL-10, LT-a, IL17A, sFasL, CXCL10, MCSF,	TGF-β, IL-1β, IL-1α, TNF, IFN-α, IL-2, IL-12, IFN-c, IL-4, IL-13, IL-5, IL-15, IL-7, IL-13, IL-9, GMCSF, LIF, CD40L, TRAIL, CCL2, CCL3, CCL4, CCL5, CCL7, CCL11, CXCL1, CXCL5, CXCL9, PDGF-BB, VEGFA, sICAM-1, VCAM-1, TGF-α, FGFb, bNGF, HGF, SCF, GCSF
Neu et al. 2014 (131)	Fukuda et al. 1994 (71)	Serum	samples collected after 2nd night of polysomnography (indwelling cannula)	early morning	−20°C	Multiplex	BD Biosciences	CBA Human flex-set kit	IL-1β, TNF, IL8, IL-10	IL-6, IFNc	
Nakatomi et al. 2014 (8)	Fukuda et al. 1994 (71) and ICC 2011	Serum	–	–	−80°C	–	analyzed by the Mitsubishi Chemical Mediance Corps	–			IL-1β, IL-6, TNF, IFN-c
Garcia et al. 2014 (132)	Fukuda et al. 1994 (71)	Serum	x	–	–	Multiplex	Millipore	–	IL-6, IL-2, IL12, IFN-c, GMCSF, CXCL10		IL-1β, IL-1α, IL-6, TNF, IL-8, IL-10, IFN-α, IL-4, LT-a, IL-5, IL-7, CCL2, CCL3, CCL4
Nakamura et al. 2013 (133)	Fukuda et al. 1994 (71)	Plasma	venous sampling throughout two nights, twice asleep and once awake (indwelling cannula)	1:00, 3:00, 5:00, 8:00 a.m.	−80°C	Multiplex	Millipore	Milliplex human multicytokine detection system			IL-1β, IL-6, TNF, IL-8, IL-10, IL-4
Maes et al. 2013 (134)	Fukuda et al. 1994 (71)	Plasma	fasting blood samples	8:30–11:30 a.m.	–	ELISA	R&D, GE Healthcare UK Ltd.	Quantikine Human TNF-α Immunoassay; Amersham Interleukin-1 alpha ((h) IL-1α); Amersham Interleukin-1 beta ((h) IL-1β)	IL-1β, IL-1α, IFN-α		
Lattie et al. 2012 (135)	Fukuda et al. 1994 (71)	Plasma	x	11:00 a.m.−3:00 p.m.	−80°C	ELISA	Quansys Biosciences and R&D	Q-Plex Human Cytokine Screen; assayed in duplicate with R&D standard	IL-1β, IL-6		TNF, IL-10, IL-2
Smylie et al. 2013 (136)	Fukuda et al. 1994 (71)	Plasma	blood drawn 3x during exercise challenge	–	−80°C	ELISA	Quansys Biosciences	Q-Plex Human Cytokine Screen (16-plex)	Males: IL-2, IL23		Females: IL-1b, IL-1α, IL-6, TNF, IL-8, IL-10, IL-2, IL-12, IFN-c, IL-4, IL-13, TNF-β, IL-5, IL-23, IL-17, IL-15; Males: IL-1b, IL-1α, IL-6, TNF, IL-8, IL-10, IL-12, IFN-c, IL-4, IL-13, TNF-β, IL-5, IL-17, IL-15
Broderick et al. 2012 (137)	ICD (Reeves et al. 2005 (231) and Fukuda et al. 1994 (71))	Plasma	fasting blood samples	Morning	−80°C	ELISA	Quansys Biosciences	Q-Plex Human Cytokine Screen (16-plex)	IL-8	IL-23	IL-1b, IL-1α, IL-6, TNF, IL-10, IFN-α, IL-2, IL-12, IFN-c, IL-4, IL-13, TNF-β, IL-5, IL-17, IL-15
Maes et al. 2012 (138)	Fukuda et al. 1994 (71)	Plasma	fasting blood samples	8:30–11:30 a.m.	–	ELISA	R&D, GE Healthcare UK Ltd.	Quantikine Human TNF-α Immunoassay; Amersham Interleukin-1 alpha ((h) IL-1α); Amersham Interleukin-1 beta ((h) IL-1β)	IL-1β, IL-1α, TNF		
Nas et al. 2011 (139)	Fukuda et al. 1994 (71)	Serum	–	–	–	ELISA	DPC Immulite 1,000 Chemistry Analyzer	IMMULITE 10,00 analyzers, kits not specified	IL-6		IL-8
White et al. 2010 (140)	Fukuda et al. 1994 (71)	Plasma	blood samples at baseline, 0.5, 8, 24, and 48 h post exercise	–	−80°C	Multiplex	Developed at the ARUP Institute for Clinical and Experimental Research (Salt Lake City, UT)	–			IL-1β, IL-6, TNF, IL-8, IL-10, IL-2, IL-12, IFN-c, IL-4, IL-13
Nakamura et al. 2010 (141)	Fukuda et al. 1994 (71)	Plasma	venous sampling throughout the night while asleep (indwelling cannula)	1:00, 3:00, 5:00, 8:00 a.m.	−80°C	Multiplex	Millipore	Beadlyte human multicytokine detection system 2			IL-1β, IL-6, TNF, IL-8, IL-10, IL-4
Nijs et al. 2010 (142)	Fukuda et al. 1994 (71)	Plasma	blood samples taken before and 1 h after exercise	–	–	ELISA	Amersham Biosciences Europe GmbH, Pierce Biotechnology Inc.	Biotrak Easy ELISA RPN5971, Endogen Human IL-1β ELISA kit			IL-1β
Robinson et al. 2010 (143)	Fukuda et al. 1994 (71)	Plasma	blood sampled at rest, at point of exhaustion, and 24 h post exercise (indwelling cannula); after overnight fast	–	–	ELISA	BD Biosciences	OptEIA			IL-6
Scully et al. 2010 (144)	Fukuda et al. 1994 (71)	Plasma	x	–	−80°C	Multiplex	Meso Scale Discovery	–	IL-1β, IL-6, IL-8		TNF, IL-10, IFN-c, IL-12p70, IL-13
Fletcher et al. 2009 (145)	Fukuda et al. 1994 (71)	Plasma	x	Morning	−80°C	Multiplex	Quansys Biosciences	Q-Plex Human Cytokine-Screen (16-plex)	IL-1β, IL-1α, IL-6, IL-12, IL4, IL-5	IL-8, TNF-β, IL-15	TNF, IL-10, IL-2, IFN-c, IL-13, IL-23, IL-17
Jammes et al. 2009 (146)	Fukuda et al. 1994 (71)	Plasma	sampling throughout exercise protocol (indwelling cannula)	–	–	ELISA	R&D	Quantikine HS Human IL-6 Immunoassay D6050; Quantikine HS Human TNF-α DTA00C			IL-6, TNF
Nater et al. 2008 (147)	Fukuda et al. 1994 (71)	Plasma	fasting blood samples taken 30 minutes after indwelling cannula was placed	7:30 a.m.	−80°C	ELISA	R&D	Quantikine HS Human IL-6 Immunoassay			Il-6
Spence et al. 2008 (148)	Fukuda et al. 1994 (71)	Plasma and Serum	x	–	−70°C	ELISA	Mercodia, Kalon Biological, R&D	–			IL-1β, TNF
Vollmer-Conna et al. 2007 (149)	Fukuda et al. 1994 (71)	PBMC, Serum	blood samples taken 1, 2, 3, 6, and 12 months after infection onset	–	−80°C	Multiplex	Bioplex, BioRad	–			IL-1β, IL-2, IL-6, IL-10, IL-12, TNF, IFN-c
Kennedy et al. 2004 (150)	Fukuda et al. 1994 (71)	Platelet poor plasma	x	same time of day	–	ELISA	R&D	–	TGF-β		
White et al. 2004 (151)	Fukuda et al. 1994 (71)	Plasma	blood collected 3 days after exercise	9:30 a.m.−12:30 p.m.	–	ELISA	R&D	–	TGF-β		
Visser et al. 2001 (152)	Fukuda et al. 1994 (71)	WBC	x	–	−20°C	ELISA	Pharmingen, R&D, Biorad	method from Cheney et al. 1989 (153)			TNFα, IL-10, IL-12, IFN-c
Cannon et al. 1999 (154)	Holmes et al. 1988 (80)	Plasma	collected 24 h post exercise	9:00 a.m.	–	ELISA, radio-immunoassay (IL-1β)	R&D	–			IL-6
Buchwald et al. 1997 (155)	Fukuda et al. 1994 (71) and Holmes et al. 1988 (80)	Serum	x	–	–	ELISA	Genzyme Diagnostics	Predicta			IL-6
Bennett et al. 1997 (156)	Holmes et al. 1988 (80)	Serum	samples shipped on dry ice for 1 year before analysis	–	−20°C	Bioassay	R&D (IL-4-dependent HT-2 cell proliferation bioassay)	–	TGF-β		
MacDonald et al. 1996 (157)	Holmes et al. 1988 (80)	Serum	x	7:00–10:00 a.m.	–	ELISA	CCC (158)	For TGFβ: specially developed in a co-investigators lab; others not specified			TGF-β, IL-1β, IL-6, TNF
Swanink et al. 1996 (159)	Sharpe et al. 1991 (228)	WBC, Serum (TGFβ)	x	8:30–11:00 a.m.	–	ELISA	R&D, Endogen	Measured as previously described in Drenth et al. 1995 (160); Quantikine (TGFβ)			TGF-β, IL-1β, IL-1α, TNF
Peterson et al. 1994 (161)	Holmes et al. 1988 (80)	Serum	blood collected at rest, immediately after exercise, and 40 min after exercise	–	−70°C	ELISA, bioassay (TGFβ)	R&D (ELISA and IL-4-dependent HT-2 cell proliferation bioassay)	Measured as previously described in Chao et al. 1991 (158)	TGF-β		IL-1β, IL-6, TNFα
Patarca et al. 1994 (162)	Holmes et al. 1988 (80)	Plasma	once a month for 3 months	7:30–10:30 a.m.	−20°C	ELISA	Endogen, R&D, Amersham, Genzyme	Intertest-4, Biokine	TNF		IL-1β, IL-1α, IL-6, IL-2, IL-4
Lloyd et al. 1994 (163)	RACP, 2002 (229)	Serum	blood was collected prior to, during, 15 min after, 4, 24 h post exercise (indwelling cannula)	–	−70°C	ELISA	Sucrosep; Centocor; Cistron Biotechnology; Biokine TNF	–			IL-1β, TNF, IFN-α, IFN-c
Linde et al. 1992 (164)	Holmes et al. 1988 (80)	Serum	serum collected < 7 days and 6 months after onset of mono	–	–	ELISA	T-Cell Sciences; IMMUNOtest Neopterin; Delfia; Medgenix; Quantikine R&D	–	IL-1α		IL-1β, IL-6, IFN-c
Chao et al. 1991 (158)	Holmes et al. 1988 (80)	PBMC, Serum (TGFβ)	1x a day, 5 consecutive days	8:00–9:00 a.m.	−20°C	ELISA, bioassay (TGFβ)	R&D (ELISA and IL-4-dependent HT-2 cell proliferation bioassay)	–	TGF-β		IL-1β, IL-6, TNF, IL-2, IL-4
Straus et al. 1989 (165)	Holmes et al. 1988 (80)	Serum	x	–	−20°C	ELISA	Genzyme	sent to same laboratory as in Cheney et al. 1989 (153), no specifications			IL-1β, TNF, IFN-α, IL-2, IFN-c
Cheney et al. 1989 (153)	Holmes et al. 1988 (80)	Serum	biobank samples	–	–	ELISA	Genzyme	sent to Specialty Laboratories, LA, no specifications	IL-2		

*The articles compared in the table include the studies reviewed by Blundell et al. (104), as well as studies published since then (distinguished by the horizontal double line in the table). Stringer et al. (166) was not reviewed by Blundell et al. (104) but is included in the table. The newer studies were found by searching “myalgic encephalomyelitis,” “chronic fatigue syndrome,” or “myalgic encephalomyelitis/chronic fatigue syndrome” with “cytokine.” Studies were selected if they included an ME/CFS group and used a cytokine assay. Though not a systematic literature review, the studies in the table serve to show the variance in methodology (from sample collection and storage to assay selection) and reported results across cytokine studies. –, not specified/reported; x, no specifications of note for sample collection; CCC, Canadian Consensus Criteria; ICC, International Consensus Criteria; ICD, International Case Definition; RACP, Royal Australasian College of Physicians; SEID, Systemic Exertion Intolerance disease*.

The corrected Table A1, Cytokine studies of ME/CFS appears below.

The authors apologize for this error and state that this does not change the scientific conclusions of the article in any way. The original table in the article has been updated.

