# Corrigendum: Neuroinflammation and Cytokines in Myalgic Encephalomyelitis/Chronic Fatigue Syndrome (ME/CFS): A Critical Review of Research Methods

**DOI:** 10.3389/fneur.2020.00863

**Published:** 2020-09-17

**Authors:** Michael B. VanElzakker, Sydney A. Brumfield, Paula S. Lara Mejia

**Affiliations:** Division of Neurotherapeutics, Massachusetts General Hospital, Harvard Medical School, Boston, MA, United States

**Keywords:** myalgic encephalomyelitis, neuroimaging, glia, microglia, PBR28, cytokines, translocator protein, positron emission tomography

In the original article, there were some minor mistakes in Table A1. *Cytokine studies of ME/CFS* as published. We have added clarifying or corrective details to several papers listed in the table, and we have added text to the figure legend to clarify its organization. We have corrected instances in which Blundell et al. (104) reported some studies' cytokine findings as “no significant difference,” when the original paper had actually reported those cytokines as below detection level. We have corrected instances in which cited papers made typographical or counting errors in their reported findings. We have added Stringer et al. (142), which was not included in the original table but should have been.

The corrected Table A1. *Cytokine studies of ME/CFS* appears below.

**Table A1:** Cytokine studies of ME/CFS.

		**Sample handling and processing**				**Assays**			**Assay results**		
**Study**	**Diagnostic criteria**	**Sample matrix**	**Collection (Specifications of note)**	**Time of collection**	**Storage**	**Method**	**Manufacturer**	**Kits**	**Increase**	**Decrease**	**No difference**
Lynn et al. 2018 (109)	CDC 1994	Serum	samples taken at 30min intervals on two consecutive days	10:00 a.m.	−80°C	Multiplex	BD Biosciences	Human CBA kit	IL-6, TNFα (response to low dose dex, LPS)	IP-10, IL-12/23p40in ME/CFS compared to healthy controls	
Richardson et al. 2018 (110)	CCC 2003	Serum	non-fasting blood samples collected after 20-min standing test	–	–	Both	BD Biosciences; activin ELISA supplied by Oxford Brookes University	Human CBA kit 560484	serum activin B		IL-2, IL-4, IL-6, IL-10, TNF, IFN γ, IL-17A, activin A
Oka et al. 2018 (111)	CDC 1994, ICC 2011, and SEID 2015 (112)	Serum and plasma (TGF-β1, BDNF)	after 8 weeks of intervention, blood sampling before and after the last session of interventional yoga	2:00–4:00.p.m	−80°C	ELISA	Fujirebio, R&D, pbl assay science, BioSource Europe S.A.; R&D	IL-6 CLEIA cartridge; Quantikine high-sensitivity ELISA human TNF-α immunoassay; VeriKine Human Interferon Alpha Multi-Subtype Serum ELISA kit; MEDGENIX human IFNγ EASIA kit; Quantikine ELISA human TGF-β1 kit, BDNF kit		TNF-α	IL-6; IFN-α, TGF-β1, BDNF; below level of detection: IFN-γ
Moneghetti et al. 2018 (113)	CDC 1994 and ICC 2011 for PEM	Serum	fasting blood sample	morning	−80°C	Multiplex	Affymetrix	51-Plex Luminex bead kit	Increased in ME/CFS but not healthy controls following exercise: CXCL 10	Decreased in ME/CFS but not healthy controls following exercise: IL-8, CCL4, TNF-β, ICAM-1	IL-1α, IL-1β, IL-1RA, IL-2, IL-4, IL-5, IL-6, IL-7, IL-10, IL12p40, IL12p70, IL-13, IL-15, IL-17, IL-17F, IL-18, LIF, FGF-β, HGF, NGF, PDGF-BB, TGFα, TGF-β1, VEGF, G-CSF, GM-CSF, M-CSF, SCF, CCL2, CCL3, CCL5, CCL7, CXCL1, CXCL5, CXCL9, CCL11, IFN-α, IFN-β, IFN-⋎, VCAM-1, CD40L, FASL, Leptin, PAI-1, Resistin, TNF-α, TRAIL
Wyller et al. 2017 (114)	CDC 1994 and CCC 2003	Plasma	fasting blood sample, no tobacco or caffeine	7:30–9:30 a.m.	−80°C	Multiplex	Bio-Rad Laboratories	Bio-Plex Human TGF-β 3-Plex			TGF-β1, TGF-β2, TGF-β3
Roerink et al. 2017 (115)	CDC 1994	Plasma	before and after 4 weeks of treatment	morning	−80°C	Multiplex, ELISA (TGF-β)	Olink Proteomics AB; R&D	Proseek Multiplex Inflammation panel; TGF-β duo-set DY240	Increased in ME/CFS compared to healthy controls at baseline: IL-12p40, CSF-1		CD40L, CCL2, IL-7, IL-8, CCL11, IL-6, IL-10, CXCL10, CXCL9, TRAIL, TNF-β, TGF-α, TGF-β1 Below level of detection: IFN-γ, IL-1α, IL-2, IL-4, IL17A, TNF
Milrad et al. 2018 (116)	CDC 1994	Plasma	–		−80°C	Multiplex	Quansys	Q-plex Human cytokine screen	Higher levels of cortisol predicted higher levels of IL-2, IL-6, TNF-α		
Montoya et al. 2017 (117)	CDC 1994	Serum	–	8:30 a.m. −3:30 p.m.	−80°C	Multiplex	Affymetrix	51-multiplex array	TGF-β; IL-13 in severe group (when stratified by severity); leptin in mild group; significant upward linear trend across severity: CCL11, CXCL1, CXCL10, G-CSF, GM-CSF, IFN-γ, IL-4, IL-5, IL-7, IL-12p70, IL-13, IL-17F, leptin, LIF, NGF, SCF, TGF-α	resistin in mild and severe groups; significant nonlinear inverted trend: ICAM1, resistin	CCL2, CCL3, CCL4, CCL5, CCL7, CD40L, CXCL5, CXCL9, FASL, FGF-basic, HGF, IFN-α, IFN-β, IL-1RA, 1L-1α, IL-1β, IL-2, IL-6, IL-8, IL-10, IL-12p40, IL-15, IL-17, M-CSF, PAI-1, PDGF-BB, TNF-a, TNF-β, TRAIL, VCAM1, VEGF
Nagy-Szakal et al. 2017 (118)	CDC 1994 and/or CCC 2003	Plasma	shipped from clinical sites	–	−80°C	Multiplex	Affymetrix	Customized Procarta immunoassay (61-plex)			IL1α, IL1β, IL1RA, IL18, IL2, IL4, IL7, IL9, IL13, IL15, IL5, IL6, LIF, IL31, IL10, IL21, IL22, IL12p40, IL12p70, IL23, IL27, IL17A, IL17F, IFNα2, IFNβ, IFNγ, TNFα (TNFSF2), TNFβ (TNFSF1), sFasL (TNFSF6), TRAIL (TNFSF10), CCL2 (MCP1), CCL3 (MIP1a), CCL4 (MIP1b), CCL5 (RANTES), CCL7 (MCP3), CCL11
											(eotaxin), CXCL1 (GROa), CXCL8 (IL8), CXCL9 (MIG), CXCL10 (IP10), CXCL12a (SDF1a), PDGFBB, VEGFA, VEGFD, sICAM1 (CD54), VCAM1 (CD106), serpin E1 (PAI1), leptin, resistin, TGFα, TGFβ, FGFβ, βNGF, HGF, SCF, MCSF (CSF1), GMCSF(CSF2), GCSF (CSF3), PlGF1, EGF, BDNF
Hornig et al. 2017 (119)	CDC 1994 and/or CCC 2003	CSF	CSF samples from biobank	–	−80°C	Multiplex	Affymetrix	Customized Procarta immunoassay (51-plex)	FGFb in Classical-ME/CFS-short duration compared to Atypical-ME/CFS-short duration; SCF in Atypical-ME/CFS compared to Classical-ME/CFS irrespective of illness duration	IL1β, IL5, IL7, IL13, IL17A, IFNα2, IFNγ, TNFα, TRAIL (TNFSF10), CCL2, CCL7, CXCL5, CXCL9, CSF3 (GCSF), βNGF, resistin, serpin E1 (PAI1) in Atypical-ME/CFS-short duration compared to Classical-ME/CFS-short duration;IL7, IL17A, CXCL9, serpin E1 in Atypical-ME/CFS-short duration compared to Classical-ME/CFS-long duration;IL5, IL13, IL17A, CXCL9, in Atypical-ME/CFS-long duration compared to Classical-ME/CFS-short duration; IL6,	IL1ra, IL1α, IL2, IL4, CXCL8 (IL8), IL10, IL12p40, IL12p70, IL15, IL17F, TNFβ, CD40L, sFasL, CCL3 (MIP1α), CCL4 (MIP1β), CCL5 (RANTES), CCL11 (eotaxin), CXCL1 (GROα), CXCL10 (IP10), TGFα, TGFβ, CSF1 (MCSF), CSF2 (GMCSF), PDGFBB, HGF, VEGFA, LIF, leptin, sICAM1 (CD54), VCAM1 (CD106)
										IL17A in Atypical-ME/CFS-long duration compared to Classical-ME/CFS-long duration	
Hanevik et al. 2017 (120)	CDC 1994	Stimulated PBMC culture	fasting blood samples	8:00–9:00 a.m.	−80°C	Multiplex	Bio-Rad Laboratories	Bioplex assays, kits not specified			IFN-γ, TNF-α, IL-1β, IL-2, IL-4, IL-6, IL-9, IL-10, IL-13, IL-17A, IL-22, MIP-1α, MIP-1β, TGFβ1, TGFβ2, TGFβ3, GM-CSF
Lidbury et al. 2017 (121)	CCC 2003	Serum	non-fasting samples collected after 20-min standing test	–	–	Both	BD Biosciences; activin kit supplied by Oxford Brookes University	Human CBA kit 560484	Activin B		IL-2, IL-4, IL-6, IL-10, activin A, follistatin; below levels of detection: IL-17A, IFN-γ, TNF-α
Milrad et al. 2017 (122)	CDC 1994	Plasma	x	11:00 a.m. −3:00 p.m.	−80°C	Multiplex	Quansys Biosciences	Q-plex Human cytokine screen	IL-1β, IL-6, TNF-a within CFS patients associated with poor sleep quality		
Lunde et al. 2016 (123)	CDC 1994 and CCC 2003	Serum	x	–	−80°C	ELISA	R&D; Invitrogen/Life technologies	BAFF and APRIL kits	BAFF, APRIL (baseline relative to healthy controls); BAFF, APRIL (post-intervention relative to baseline)		
Huth et al. 2016 (124)	CDC 1994	PBMC	x	7:30–10:00 a.m.	–	Neither	BD Biosciences; Biolegend	intracellular staining of stimulated and unstimulated PBMC cultures			IFN-γ, TNF-α and GM-CSF increased in culture after challenge, but no difference between groups
Russell et al. 2016 (125)	Jason revision for pediatrics of CDC 1994 and ICD, Reeves et al. 2005 (126)	Plasma	fasting blood sample	morning	−80°C	Multiplex	Quansys Biosciences	Q-plex Human cytokine screen (16-plex)	IL-4, IL-5, IL-12, LTα in 50+ y.o. ME/CFS females relative to healthy controls; IL-8 in ME/CFS adolescent females relative to healthy controls	IL-8, IL-15 in 50+ y.o. ME/CFS females relative to healthy controls; IL-23 in ME/CFS adolescent females relative to healthy controls	IL-1α, IL-1β, IL-2, IL-6, IL-10, IL-13, IL-17, IFNγ, TNFα
**Study**	**Diagnostic criteria**	**Sample matrix**	**Collection (Specifications of note)**	**Time of collection**	**Storage**	**Method**	**Manufacturer**	**Kits**	**Increase**	**Decrease**	**No difference**
Landi et al. 2016 (127)	CDC 1994 and/or CCC 2003	Plasma	samples from Solve ME/CFS BioBank	–	−80°C	Multiplex	Meso Scale Discovery	MSD Human V-PLEX Plus Kits: Chemokine Panel 1, Cytokine Panel 1, and Pro-inflammatory Panel 1; Human Eotaxin-2 Kit, a custom-designed 3-Plex kit, a custom-designed 1-Plex kit	CCL24 univariate analysis	IL-16, IL-7, VEGF-A, CXCL9, CX3CL1 univariate analysis; IL-16, IL-7, VEGF-A by multivariate cluster analysis	IL-17A, TNFβ, CCL19, CCL11, IL-1β, TNFα, CCL3, CCL17, CCL2, IFN-g, IL-15, CCL26, IL-6, IL-12/23p40, CCL22, IL-5, CCL13, IL-1α, CCL4, GM-CSF, IL-10, IL-4, IL-13, IL-2, CXCL10, IL-12p70, IL-8, B2M
Hardcastle et al. 2015 (128)	CDC 1994	Serum	non-fasting blood sample	8:30–11:30 a.m.	–	Multiplex	BioRad	BioPlex Pro human cytokine 27-plex	IL-1β in moderate compared to severe ME/CFS; RANTES in moderate compared to severe ME/CFS and healthy controls; IFN-γ in severe compared to moderate ME/CFS; IL-7, IL-8 in severe compared to moderate ME/CFS and healthy controls	IL-6 in moderate compared to severe ME/CFS and healthy controls	IL-1ra, IL-2, IL-4, IL-5, IL-6, IL-9, IL-10, IL-12p70, IL-13, IL-17, FGF, eotaxin, G-CSF, GM-CSF, IP-10, PDGF-BB, TNF-α, VEGF, MCP1, MIP1a, and MIP1b
Peterson et al. 2015 (129)	CDC 1994	CSF	CSF samples via lumbar puncture	–	−80°C	Multiplex	BioRad	BioPlex Pro human cytokine 27-plex		IL-10	IL-1rα, IL-2, IL-6, IL-7, IL-8, IL-9, IL-12p70, IL-13, IL-15, IL-17, basic FGF, eotaxin, G-CSF, GM-CSF, IFN-γ, IP-10, MCP-1, RANTES, TNF-α, and PDGF-BB Below limit of detection: IL-1β, IL-4, MIP-1α, MIP-1β, IL-5 (VEGF listed in multiplex section)
**Study**	**Diagnostic criteria**	**Sample matrix**	**Collection (Specifications of note)**	**Time of collection**	**Storage**	**Method**	**Manufacturer**	**Kits**	**Increase**	**Decrease**	**No difference**
Khaiboullina et al. 2015 (130)	CDC 1994 or Carruthers et al. 2011, 2003 (131, 132)	Serum	some blood samples shipped overnight	–	−80°C	Multiplex	Bio-Rad Laboratories	Bio-Plex Human Cytokine 27-Plex Panel, Bio-Plex Pro Human Chemokine Panels (40-plex), Bio-Plex Pro Human Th17 Cytokine Panels, Bio-Plex Cytokine 21-Plex Panels	CCL1, CCL2, CCL20, CCL3, CXCL10, IFN-γ, IL-1, IL-10, IL13, IL-1β, IL25, IL-31, IL-4, IL-6, IL-7, IL12 (p75), TNF-α	CCL11, CCL17, CCL19, CCL21, CCL25, CCL26, CCL3, CCL4, CCL5, CCL8, CSF1, CSF3, CX3CL1, CXCL1, CXCL13, CXCL6, CXCL8, HGF, IL-17F, IL5, IL-9, LIF, MIF, PDGF, TRAIL, VEGF	CCL13, CCL22, CCL23, CCL24, CCL27, CCL7, CXCL11, CXCL12a, CXCL12ab, CXCL16, CXCL2, CXCL5, CXCL9, FGF, GMCSF, IFN-α, IL-12 (p40), IL-15, IL-16, IL17A, IL-18, IL-1RA, IL-1α, IL-2, IL-21, IL-22, IL-23, IL-3, IL-33, IL-2RA, sCD40L, SCF, SCGF-β, TNF-β, β-NGF
Wyller et al. 2015 (133)	Royal College of Pediatrics & Child Health (2004) (134); National Institutes of Health and Clinical Excellence (2007) (135)	Plasma	fasting blood samples; abstained from tobacco and caffeine for 48 h	7:30–9:30 a.m.	−80°C	Multiplex	Bio-Rad Laboratories	Bio-Plex Human Cytokine 27-Plex Panel			IL-1β, IL-1RA, IL-2, IL-4, IL-5, IL-6, IL-7. IL8, IL-9, IL-10, IL-12, IL-13, IL-17, IFN-γ, CCL2, CCL3, CCL4, CCL5, CXCL10, PDGF-BB, VEGF, FGF, TNF
Hornig et al. 2015 (136)	CDC 1994, CCC 2003, or Jason et al. 2010 (137)	Plasma	samples shipped overnight	10:00 a.m. −2:00 p.m.	−80°C	Multiplex	Affymetrix	customized Procarta immunoassay	Leptin	IL-6, IL-8, IL-10, LT-α, IL17A, sFasL, CXCL10, M-CSF	TGF-β, IL-1β, IL-1α, TNF-α, IFN-α2, IL-2, IL-12p40, IL-12p70, IFN-c, IL-4, IL-13, IL-5, IL-15, IL-7, GMCSF, LIF, CD40L, TRAIL, CCL2, CCL3, CCL4, CCL5, CCL7, CCL11, CXCL1, CXCL5, CXCL9, PDGF-BB, VEGFA, sICAM-1, VCAM-1, TGF-α, FGFb, bNGF, HGF, SCF, G-CSF, IL17F, IFN-B, serpin E1, resistin
Neu et al. 2014 (138)	CDC 1994	Serum	samples collected after 2nd night of polysomnography (indwelling cannula)	early morning	−20°C	Multiplex	BD Biosciences	CBA Human flex-set kit	IL-1β, TNF-α, IL8, IL-10	IL-6, IFN-γ	
**Study**	**Diagnostic criteria**	**Sample matrix**	**Collection (Specifications of note)**	**Time of collection**	**Storage**	**Method**	**Manufacturer**	**Kits**	**Increase**	**Decrease**	**No difference**
Nakatomi et al. 2014 (8)	CDC 1994 and Carruthers	Serum	–	–	−80°C	–	analyzed by the Mitsubishi Chemical Mediance Corps	–			IL-1β, IL-6, TNF-α, IFN- γ
Garcia et al. 2014 (139)	CDC 1994	Serum	x	–	–	Multiplex	Millipore	–	IL-6, IL-2, IL12p70, IFN-c, GMCSF, CXCL10		IL-1β, IL-1α, TNF-α, IL-8, IL-10, IFN-γ, IL-4, LT-α, IL-5, IL-7, CCL2, CCL3, CCL4, IL-3
Nakamura et al. 2013 (140)	CDC 1994	Plasma	venous sampling throughout 3 nights of different sleep conditions: normal, after exercise testing, and without sleep (indwelling cannula)	1 h after cannula placement (awake), 1:00, 3:00, 5:00, between 7:15 a.m.−8:00 a.m. after waking	−80°C	Multiplex	Millipore	Milliplex human multicytokine detection system			IL-1β, IL-6, TNF-α, IL-8, IL-10, IL-4
Maes et al. 2013 (141)	CDC 1994	Plasma	fasting blood samples	8:30–11:30 a.m.	–	ELISA	R&D, GE Healthcare UK Ltd.	Quantikine Human TNF-α Immunoassay; Amersham Interleukin-1 alpha [(h) IL-1α]; Amersham Interleukin-1 beta [(h) IL-1β]	IL-1β, IL-1α, TNF-α		
Stringer et al. 2013 (142)	CDC 1994	Serum	25 consecutive days of blood draws, site of blood draw rotated regularly	Visits were held within a 2 h window	−80°C	Multiplex	Affymetrix	Human 51-plex Luminex	Leptin predicted daily fatigue severity in ME/CFS patients but not in healthy controls		ICAM1, TGF-b, IL10, PDGFBB, MCP3, IL1B, ENA78, MCSF, IFN-a, IL12P40, FGF-b, RANTES, TNF-b, IL1RA, PAI1, FASL, VCAM1, MCP1, IP10, HGF, CD40L, IFN-g, IL2, VEGF, GMCSF, MIP1B, SCF, TNF-a, TRAIL, IL5, MIP1A, MIG, GCSF, RESISTIN, IL7, EOTAXIN, TGF-a, IL17F, IL15, IFN-g, IL13, IFN-b, IL12P70, IL6, IL4, NGF, IL1A, LIF, IL8, GRO-a
**Study**	**Diagnostic criteria**	**Sample matrix**	**Collection (Specifications of note)**	**Time of collection**	**Storage**	**Method**	**Manufacturer**	**Kits**	**Increase**	**Decrease**	**No difference**
Lattie et al. 2012 (143)	CDC 1994	Plasma	x	11:00 a.m. −3:00 p.m.	−80°C	ELISA & Multiplex	Quansys Biosciences and R&D	Q-Plex Human Cytokine Screen; assayed in duplicate with R&D standard	IL-1β, IL-6		TNF-α, IL-10, IL-2
Smylie et al. 2013 (144)	CDC 1994 and Reeves et al. 2005 (126)	Plasma	blood drawn 3x during exercise challenge: at rest (T0), at VO_2_ max (T1), 4 h after exercise (T3)	–	−80°C	Multiplex	Quansys Biosciences	Q-Plex Human Cytokine Screen (16-plex)	Males: IL-2 (T0, T1, T2), IL23 (T0, T2); Females: IL-1α (T1)	Females: IL-4 (T1)	Females: IL-1b, IL-6, TNF-α, IL-8, IL-10, IL-2, IL-12, IFN-c, IL-13, TNF-β, IL-5, IL-23, IL-17, IL-15; Males: IL-1b, IL-1α, IL-6, TNF, IL-8, IL-10, IL-12, IFN-c, IL-4, IL-13, TNF-β, IL-5, IL-17, IL-15
Broderick et al. 2012 (145)	Reeves et al. 2005 (126) and Jason et al. 2006 (146)	Plasma	fasting blood samples	morning	−80°C	Multiplex	Quansys Biosciences	Q-Plex Human Cytokine Screen (16-plex)	IL-8	IL-23	IL-1β, IL-1α, IL-6, TNF-α, IL-10, IFN-α, IL-2, IL-12p70, IL-4, IL-13, TNF-β, IL-5, IL-17, IL-15
Maes et al. 2012 (147)	CDC 1994	Plasma	fasting blood samples	8:30–11:30 a.m.	–	ELISA	R&D, GE Healthcare UK Ltd.	Quantikine Human TNF-α Immunoassay; Amersham Interleukin-1 alpha [(h) IL-1α]; Amersham Interleukin-1 beta [(h) IL-1β]	IL-1β, IL-1α, TNF-α		
Nas et al. 2011 (148)	ICC 2011	Serum	–	–	–	–	DPC Immulite 1000 Chemistry Analyzer	IMMULITE 1000 analyzers, kits not specified	IL-6, IL2r		IL-8
White et al. 2010 (149)	CDC 1994	Serum	blood samples at baseline, 0.5, 8, 24, and 48 h post exercise	–	−80°C	Multiplex	Developed at the ARUP Institute for Clinical and Experimental Research (Salt Lake City, UT)	–		CD40L	IL-1β, IL-6, TNF-α, IL-8, IL-10, IL-2, IL-12, IFN-γ, IL-4, IL-13
Nakamura et al. 2010 (150)	CDC 1994	Plasma	venous sampling throughout the night while asleep (indwelling cannula)	1:00, 3:00, 5:00, 7-7:30 a.m.	−80°C	Multiplex	Millipore	Beadlyte human multicytokine detection system 2	IL-10 in CFS without FM compared to controls at 3:00am and 5:00am, and compared to CFS with FM at 5:00am		IL-1β, IL-6, TNF, IL-8, IL-4
**Study**	**Diagnostic criteria**	**Sample matrix**	**Collection (Specifications of note)**	**Time of collection**	**Storage**	**Method**	**Manufacturer**	**Kits**	**Increase**	**Decrease**	**No difference**
Nijs et al. 2010 (151)	CDC 1994	–	blood samples taken before and 1 h after exercise	–	–	ELISA	Amersham Biosciences Europe GmbH, Pierce Biotechnology Inc.	Biotrak Easy ELISA RPN5971, Endogen Human IL-1β ELISA kit			Below level of detection: IL-1β
Robinson et al. 2010 (152)	CDC 1994	Plasma	blood sampled at rest, at point of exhaustion, and 24 h post exercise (indwelling cannula); after overnight fast and abstaining from alcohol, caffeine, and strenuous activity for 24 h	–	−80°C	ELISA	BD Biosciences	OptEIA			IL-6
Scully et al. 2010 (153)	CDC 1994	Plasma	x	–	−80°C	Multiplex	Meso Scale Discovery	–	IL-1β, IL-6, IL-8		TNF-α, IL-10, IFN-γ, IL-12p70, IL-13
Fletcher et al. 2009 (154)	CDC 1994, Reeves et al. 2005 (126)	Plasma	x	morning	−80°C	Multiplex	Quansys Biosciences	Q-Plex Human Cytokine-Screen (16-plex)	IL-1β, IL-1α, IL-6, IL-12, IL-4, IL-5, TNF-β	IL-8, IL-15, IL-13	TNF-α, IL-10, IL-2, IFN-γ, IL-13, IL-23, IL-17
Jammes et al. 2009 (155)	CDC 1994	Plasma	sampling throughout exercise protocol (indwelling cannula)	–	–	ELISA	R&D	Quantikine HS Human IL-6 Immunoassay D6050; Quantikine HS Human TNF-α DTA00C		Depressed IL-6 and TNF-α response to exercise in CFS (absence of significant post-exercise increase as in controls)	Baseline IL-6 and TNF-α
Nater et al. 2008 (156)	CDC 1994	Plasma	fasting blood samples taken 30 minutes after indwelling cannula was placed	7:30 a.m.	−80°C	ELISA	R&D	Quantikine HS Human IL-6 Immunoassay	lL-6 (not significant after controlling for BMI, even though BMI did not differ across groups)		
**Study**	**Diagnostic criteria**	**Sample matrix**	**Collection (Specifications of note)**	**Time of collection**	**Storage**	**Method**	**Manufacturer**	**Kits**	**Increase**	**Decrease**	**No difference**
Spence et al. 2008 (157)	CDC 1994	Serum	x	same time of day	−70°C	ELISA	R&D	–			IL-1, TNF-α
Vollmer-Conna et al. 2007 (158)	CDC 1994	Serum	blood samples taken 1, 2, 3, 6, 12 months after infection onset	–	−80°C	Multiplex	BioRad	Bioplex			IL-1β, IL-2, IL-4, IL-6, IL-10, IL-12, TNF-α, IFN-γ; serum cytokine levels almost exclusively below detection level
Kennedy et al. 2004 (159)	CDC 1994	Platelet poor plasma	x	same time of day	–	ELISA	R&D	–	TGF-β1		
White et al. 2004 (160)	CDC 1994	Plasma	blood collected at baseline, immediately pre- & post-exercise, 3 h and 3 days post-exercise	9:30 a.m. −12:30 p.m.	–	ELISA	R&D	–	TGF-β1 at all time points compared to healthy controls; TNF-α at 3 h and 3 days post-exercise compared to healthy controls		IL-1-α
Visser et al. 2001 (161)	CDC 1994	WBC	fasting blood samples	7:00 a.m. −10:00 a.m.	−20°C	ELISA	Pharmingen, R&D, Biorad	method from Cheney et al. 1989 (162)			TNFα, IL-10, IL-12, IFN-c
Cannon et al. 1999 (163)	CDC 1988	Plasma	collected 24 h post exercise	9:00 a.m.	–	ELISA	R&D	–			IL-6 (majority of samples below detection level)
Buchwald et al. 1997 (164)	CDC 1988 and 1994	Serum	x	–	–	ELISA	Genzyme Diagnostics	Predicta			IL-6
Bennett et al. 1997 (165)	CDC 1988	Serum	samples shipped on dry ice 1 year before analysis	–	−20°C	Bioassay	R&D (IL-4-dependent HT-2 cell proliferation bioassay)	–	TGF-β		
MacDonald et al. 1996 (166)	CDC 1988	Serum	x	7:00–10:00 a.m.	–	ELISA	CCC (167)	For TGFβ: specially developed in a co-investigators lab; others not specified			TGF-β, IL-1β, IL-6, TNF-α
**Study**	**Diagnostic criteria**	**Sample matrix**	**Collection (Specifications of note)**	**Time of collection**	**Storage**	**Method**	**Manufacturer**	**Kits**	**Increase**	**Decrease**	**No difference**
Swanink et al. 1996 (168)	Sharpe et al. 1991 (169)	Plasma, Serum (TGFβ)	no caffeine for 48 h; stopped all medications prior to blood draw	8:30–11:00 a.m.	–	ELISA	R&D, Endogen	Measured as previously described in Drenth et al. 1995 (170); Quantikine (TGFβ)			TGF-β, IL-1β, IL-1α, TNF
Peterson et al. 1994 (171)	CDC 1988 and Schluederberg et al. 1992 (172)	Serum	blood collected at rest, immediately after exercise, and 40 min after exercise	–	−70°C	ELISA, bioassay (TGFβ)	R&D (ELISA and IL-4-dependent HT-2 cell proliferation bioassay)	Measured as previously described in Chao et al. 1991 (167)	TGF-β elevated in CFS compared to healthy controls at rest and 40 min after exercise		Below limit of detection at all time points in CFS and healthy controls: IL-1β, IL-6, TNFα
Patarca et al. 1994 (173)	CDC 1988	Plasma and serum	once a month for 3 months	7:30–10:30 a.m.	−20°C	ELISA	Endogen, R&D, Amersham, Genzyme, T-Cell Diagnostics	Intertest-4, Biokine	TNF-α, TNF-β		IL-1β, IL-1α, IL-6, IL-2, IL-4, GMCSF
Lloyd et al. 1994 (174)	Lloyd 1988 and Holmes 1988	Serum	blood was collected prior to, during, 15 min after, 4, 24 h post exercise (indwelling cannula)	–	−70°C	ELISA, radioimmunoassay	Sucrosep; Centocor; Cistron Biotechnology; T Cell Sciences	Biokine TNF			IL-1β, TNF-α, IFN-α, IFN-γ: all cytokines at all time points were at or below the level of detection; no significant differences between CFS and healthy controls
Linde et al. 1992 (175)	CDC 1988	Serum	blood samples drawn >1 year after onset of symptoms & during a period of severe clinical symptoms (samples stored at room temperature for 2 h)	–	–	ELISA, radioimmunoassay, dissociation-enhanced lanthanide fluoroimmunoassay, EASIA	Delfia; RIA; Medgenix; Quantikine R&D	–	In CFS patients and acute infectious mononucleosis patients compared to controls: IL-1α		IL-1β, IL-6; IFN-γ (no difference between CFS patients and controls, but increased in acute infectious mononucleosis patients compared to controls); IFN-α: many samples below level of detection
**Study**	**Diagnostic criteria**	**Sample matrix**	**Collection (Specifications of note)**	**Time of collection**	**Storage**	**Method**	**Manufacturer**	**Kits**	**Increase**	**Decrease**	**No difference**
Chao et al. 1991 (167)	CDC 1988	Serum	1x a day, 5 consecutive days; no anti-inflammatory drugs for at least 3 days prior to blood sampling	8:00–9:00 a.m.	−20°C	ELISA, bioassay (TGFβ)	R&D (ELISA and IL-4-dependent HT-2 cell proliferation bioassay)	–	TGF-β		IL-1β, IL-6, TNF: most samples had levels below detection limit and did not differ significantly between groups; IL-2, IL-4: levels below detection limit
Straus et al. 1989 (176)	CDC 1988	Serum, plasma (TNF)	samples shipped on dry ice	–	−20°C	ELISA, radioimmunoassay	–	sent to same laboratory as in Cheney et al. 1989 (162), no specifications			IL-1β, IFN-α, IFN-γ: most samples had levels below detection limit, no significant difference between groups; TNF, IL-2
Cheney et al. 1989 (162)	CDC 1988	Serum	samples were shipped from geographically separate regions	–	–	ELISA	Genzyme	sent to Specialty Laboratories, LA, no specifications	IL-2		

*Research articles compared in this table include the studies reviewed by Blundell et al. (104), as well as studies published since then (distinguished by the horizontal double line in the table). The newer studies were found by searching for “cytokine” along with “myalgic encephalomyelitis,” “chronic fatigue syndrome,” “myalgic encephalomyelitis/chronic fatigue syndrome,” relevant abbreviations, or a combination of the abbreviations. Studies were selected if they included an ME/CFS group and used a cytokine assay. Though not a complete systematic literature review, this table is intended to show the variability in methods (from sample collection and storage to assay selection) and reported results across ME/CFS cytokine studies. We use the terminology used by each paper, and report analytes that each paper defined as a cytokine. The table primarily addresses case-control studies so that methods could be compared. The table lists absolute values and does not include descriptions of multiple cytokines added to regression models. –, not specified/reported. x, no specifications of note for sample collection*.

In the original article, the reference for Hardcastle et al. (2015) was incorrectly written as Hardcastle SL, Brenu EW, Johnston S, Nguyen T, Huth T, Ramos S, et al. Longitudinal analysis of immune abnormalities in varying severities of chronic fatigue syndrome/myalgic encephalomyelitis patients. *J Trans Med*. (2015) 13:299. doi: 10.1186/s12967-015-0653-3

That citation should have instead been: Hardcastle SL, Brenu EW, Johnston S, Nguyen T, Huth T, Ramos S, et al. Serum immune proteins in moderate and severe chronic fatigue syndrome/myalgic encephalomyelitis patients. *Int J Med Sci*. (2015) 12:764–72. doi: 10.7150/ijms.12399

The authors apologize for this error and state that this does not change the scientific conclusions of the article in any way. The original article has been updated.
